# Hydration- and Temperature-Dependent Fluorescence Spectra of Laurdan Conformers in a DPPC Membrane

**DOI:** 10.3390/cells13151232

**Published:** 2024-07-23

**Authors:** Stefan Knippenberg, Kathakali De, Christopher Aisenbrey, Burkhard Bechinger, Silvio Osella

**Affiliations:** 1 Theory Lab, Hasselt University, Agoralaan Building D, 3590 Diepenbeek, Belgium; 2 Institut de Chimie de Strasbourg, University of Strasbourg/CNRS, UMR7177, rue Blaise Pascal, F-67008 Strasbourg, France; kde@unistra.fr (K.D.);; 3 Chemical and Biological Systems Simulation Lab, Centre of New Technologies, University of Warsaw, Banacha 2C, 02-097 Warsaw, Poland

**Keywords:** multiscale computational approach, lipid bilayer, hydration, fluorescence properties, laurdan

## Abstract

The widely used Laurdan probe has two conformers, resulting in different optical properties when embedded in a lipid bilayer membrane, as demonstrated by our previous simulations. Up to now, the two conformers’ optical responses have, however, not been investigated when the temperature and the phase of the membrane change. Since Laurdan is known to be both a molecular rotor and a solvatochromic probe, it is subject to a profound interaction with both neighboring lipids and water molecules. In the current study, molecular dynamics simulations and hybrid Quantum Mechanics/Molecular Mechanics calculations are performed for a DPPC membrane at eight temperatures between 270K and 320K, while the position, orientation, fluorescence lifetime and fluorescence anisotropy of the embedded probes are monitored. The importance of both conformers is proven through a stringent comparison with experiments, which corroborates the theoretical findings. It is seen that for Conf-I, the excited state lifetime is longer than the relaxation of the environment, while for Conf-II, the surroundings are not yet adapted when the probe returns to the ground state. Throughout the temperature range, the lifetime and anisotropy decay curves can be used to identify the different membrane phases. The current work might, therefore, be of importance for biomedical studies on diseases, which are associated with cell membrane transformations.

## 1. Introduction

Depending on the temperature and the composition of biological tissues, lipid bilayers have different properties and phases. Their behavior is mainly determined by order–disorder transitions, which are associated with melting phenomena of the lipid hydrocarbon chains [1]. For membranes consisting of 1,2-dipalmitoylphosphatidylcholine (DPPC), the liquid crystal phase (L_c_) is found below 280K, the gel or L_β’_ phase manifests between 290K and 305K, a so-called ripple phase (P_β’_) that is the most prominent at 310K, while at higher temperatures (above 315K), the liquid disordered or L_α_ phase can be seen [2,3]. At the latter temperatures, a strong increase in permeability is observed, which is well exploited to deliver, e.g., anticancer drugs to solid tumors in hyperthermia therapy [4,5]. Membrane phase changes are related to viscosity alterations and diseases, like atherosclerosis, Alzheimer’s or diabetes [6,7], and manifest through alterations in lipid conformational disorder and mobility, which affect the membrane structure and dynamics [8]. At the molecular scale, this transition in the saturated lipid tails boils down to the conformational conversion of the acyl chains from all trans dihedral angles to several gauche ones. Marsh’s Handbook of Lipid Bilayers reports phase transitions at 294.3K (L_c_ to L_β’_), 307.3K (L_β’_ to P_β’_) and 314.5K (P_β’_ to L_α_) [9].

To determine the characteristics of a membrane phase, the lateral diffusion coefficients of embedded fluorescent probe molecules can be measured, and their transport can be followed by techniques, like fluorescence recovery after photobleaching (FRAP) [10,11], fluorescence correlation spectroscopy (FCS) [12,13,14] and single-particle tracking [15]. Furthermore, a widely used family of probes named solvatochromic probes change their spectroscopic properties depending on the environment they are immersed in. For these probes, the interaction between the excited state dipole moment and the environment influences the optical spectra. As a result, a change in the phase of the membrane, which in turn changes its fluidity and water content, has a strong impact on the optical properties of the probe. A second class of widely used probes are molecular rotors, for which the change in conformation is enabled or rather blocked through the biological surroundings, leading to different optical responses depending on the membrane phase. For these molecules, measurements of time-resolved fluorescence and fluorescence anisotropy can be performed [16,17,18]. A classic example of a flexible molecule is, for instance, diphenylhexatriene (DPH), whose static and time-dependent optical properties depend both on the environment and its conformation. This molecular probe can be used to discriminate between membrane phases [19]. An archetype of a solvatochromic probe, which can be triggered by light (labelled as chromophore), is azobenzene. It has been shown that its trans-to-cis isomerization can be either enhanced or hampered by the membrane phase, adding a degree of control to the probes’ optical properties [20]. Conformationally versatile probes like azobenzene and its derivatives can also be covalently linked to the lipid tails, and in this case, the membrane’s structure and viscosity can even be dynamically controlled by light, and the notion of an optically controlled domain can be considered [21].

The widely used Laurdan probe has long been considered a member of the solvatochromic probes’ family [22]. Indeed, density functional theory (DFT) calculations show that its state dipole moment increases from ~7 to ~14 D after excitation [23], and that a red shift of ~50 nm is seen for the fluorescence maximum when a glycerophospholipid membrane with saturated lipid tails (such as DPPC or DPPG) undergoes an L_β’_ to L_α_ transition [24,25]. Interestingly, this shift is independent from the nature of the glycerophospholipid polar head group [25]. Through the use of the Generalized Polarization (GP) function, which is based on the variations in fluorescence intensity in different bands of wavelengths in the spectrum, Laurdan can be used to quantitatively determine the relative amount of each phase when the phases coexist in a lipid bilayer [24]. The GP value is strongly influenced by the different orientations of the emitting transition dipole moment with respect to the laboratory axis [26]. However, Laurdan’s values were not explained by just simplifying membrane differences to differences between their dielectric constants, and the hypothesis that the reorientation of water dipoles around the probe is responsible for the shift was formulated [25].

**Figure 1:**
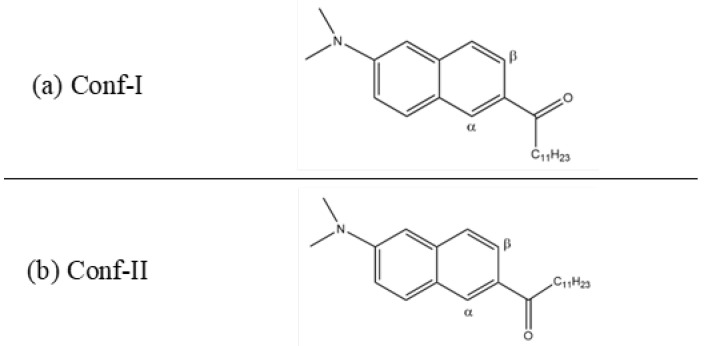
Depictions of the two conformers of Laurdan in gas phase: (**a**) Conf-I and (**b**) Conf-II. The relevant α and β positions of naphthalene are indicated.

To date, the conformational versatility with respect to the carbonyl group of the Laurdan probe on the GP measurements has not been considered. Through our simulated results in 2019, we were the first to find that this flexibility permits the use of Laurdan as a molecular rotor [27], which has since been confirmed experimentally [28]. As our studies of the probe in L_α_ and L_β’_ phases proved, the positions and orientations of the two conformers fundamentally differ [23,27,29]. For the conformer whose carbonyl oxygen points toward the *β* position of the naphthalene core (Conf-I, see Figure 1), an elongated form in its electronical ground state in DPPC (L_β’_) is observed. For the conformer with the carbonyl oxygen towards the α position (Conf-II, Figure 1), an L-shape is seen [23]. Interestingly, conformational changes are not observed in DPPC, while in DOPC, changes between the two conformations are allowed, due to the increased fluidity of the environment [23,27].

Based on two-photon excitation fluorescence microscopy, the model of water relaxation has been refined: the observation of a broad GP distribution in L_α_ relative to the L_β’_ phase was seen as proof of the existence of a large dynamical heterogeneity in the Lα phase [30]. The results were rationalized considering that the membrane contains a distribution of different cavities with different amounts of dynamically restricted water molecules in which Laurdan can reside. Although, on average, no more than two or three water molecules are found around the probe [30], for individual sites, this amount can vary. The larger the amount of solvent molecules, the lower the GP and the larger the cavity around the probe. Experiments exhibiting a similar response of the probe’s emission on bilayers with a chemical environment, which differ at the probe’s position, support the idea that water relaxation is the main cause of the emission shift [31,32]. Classical polarization spectra on Laurdan and on its variant with an isopropyl residue at the amino side (the so-called Laurisan molecule) showed that intramolecular reorientations do not affect the shift [33].

In this joint computational and experimental study, we focus on both Laurdan conformers embedded in DPPC, investigate the influence of the water layers on the probe’s conformational differences and shed light on the changes in fluorescence properties between the L_c_, L_β’_, P_β’_ and L_α_ phases. First, we give the computational details of the molecular dynamics (MD) and hybrid Quantum Mechanics/Molecular Mechanics (QM/MM) calculations and describe the performed optical experiments. In the discussion, the atomistic calculations on Conf-I and Conf-II are analysed, and insights are obtained with respect to the position and orientation of both conformers of the probe for a temperature range from 270K to 320K. Thereafter, these results are related to the simulated optical properties, while comparisons to the obtained data from the fluorescence experiments are made. Special attention is paid to the lifetime, the emission wavelength, as well as the time-dependent and steady-state fluorescence anisotropy and the role of water molecules in the proximity of the probe. Finally, the conclusions of the work are given.

## 2. Methodology

### 2.1. Computational Simulations

All presented molecular dynamics (MD) simulations were performed by means of the Gromacs 2019.1 software and the 43A1-S3 GROMOS force field [34,35], whose efficiency and performance for saturated lipids are generally well established [36]. Moreover, it has been shown that it is accurate in the description of the different phase transitions of several lipids forming molecules [35,37,38,39,40]. Then, 400 ns long excited state MD simulations were performed, in which the ground state DPPC (L_β’_) lipid bilayer system is used (as in our previous studies [23,27]), while Conf-I and Conf-II conformers of Laurdan were embedded in their optimized S_1_ excited state. This ensures the use of fully equilibrated initial conditions. Since Laurdan is both a solvatochromic probe and a molecular rotor, its properties are heavily dependent on the lipid membrane phase. ESP charges for the S_1_ excited state (see Table S6) were obtained by means of TDDFT calculations, at the CAM-B3LYP functional with the 6-31G(d) basis set and the Gaussian 09 suite of programs [41,42]. The plots and analyses presented in the current work are based on the equilibrated MD window between 240 ns and 400 ns. The lipid bilayer consists of 64 DPPC molecules per layer, is solvated with 3314 water molecules and is neutralized with sodium and chlorine ions at the physiological concentration. The TIP3P parameters were used to model the water solvent [43]. The time step for the propagation in time was set to 2 fs by means of the LINCS algorithm [44]. The particle mesh Ewald (PME) method was used to compute van der Waals and Coulomb interactions within a cutoff of 1.2 nm. An orthorhombic box of 5 nm in width, 6 nm in length and 8 nm in depth directions was considered. The *z*-axis was set to be normal to the membrane plane. Periodic boundary conditions were used along the three dimensions. The simulations were performed in the canonical NPT ensemble using the Nosé–Hoover thermostat (reference temperature specific for each simulation, with a time constant of 0.5 ps) and the anisotropic Parrinello–Rahman barostat (pressure of 1 bar, with a time constant of 5 ps and compressibility of 4.5 × 10^−5^ bar^−1^). Different production runs for each conformer were performed at temperatures of 270, 280, 290, 298, 305, 310, 315 and 320K [45,46]. The MD simulations were considered converged when the angle between the transition dipole moment of the probe and the membrane normal had fluctuations lower than 5 degrees (convergence of these simulations is depicted in Figure S1 for T = 298K).

From the 400 ns long MD simulations, 50 uncorrelated snapshots were extracted. For simplicity, a window of 100 ns was considered by sampling every 2 ns for both conformers. The followed methodology was validated and reported in detail in our previous studies [19,27,47]. Briefly, a cylindrical cutoff of 10 nm around the membrane molecules surrounding the probe was applied, as well as a hemi-spherical cutoff of 1.5 nm for water molecules in close proximity to the membrane. The extracted snapshots were considered as inputs to perform QM/MM calculations with the Dalton2016 program [48], using the electrostatic embedding scheme. The system was partitioned as follows: probe was considered at the QM level of theory, while the cylindrical environment was considered as point charges, as described at the MM level (from the snapshots selected from the MD run). Over the 50 snapshots created, QM/MM single-point calculations were performed, using the time-dependent density functional theory along with the CAM-B3LYP functional [42] and the Dunning’s cc-pVDZ basis set [49]. The three lowest energy excited states were considered in the calculations, to ensure energy convergence of the excited state energy. This functional/basis set combination was benchmarked against post-Hartree Fock methods and other density functionals for Laurdan [29] and proven accurate in a number of precedent studies of optical probes embedded in a lipid bilayer [50].

### 2.2. Sample Preparation

DPPC and 1% (mol/mol) Laurdan were co-dissolved in chloroform/methanol 2/1 (both Spectrosol grade, Carlo-Erba, Val de Reuil, France). The organic solvent was removed with a stream of nitrogen to form a thin film on a glass vial, and traces of organic solvents were removed in high vacuum overnight. A buffer of 10 mM Tris pH 7 and 100 mM NaCl was added to form a 10 mM lipid stock suspension. Multi-lamellar vesicles were produced by 4 freeze–thaw cycles. Vesicles were extruded 11 times through polycarbonate filters with a pore size of 100 nm at T = 50 °C (Whatman/cytiva, Little Chalfont, UK). For the measurement, the vesicle suspension was diluted to 1 mM lipid concentration.

### 2.3. Fluorescence Measurements

All fluorescence measurements were performed on a Fluoromax spectrometer equipped with a nanoLED pulsed diode controller for lifetime measurements (Horiba, Kyoto, Japan). A low-pass glass filter (FGL400S, Thorlabs, Newton, NJ, USA) was placed in the emission pathway to minimize light scattering artifacts. For lifetime measurements, a NanoLED-340 with a peak wavelength of 342 nm was used. Temperature was controlled with a water bath (Haake, Karlsruhe, Germany) and monitored with a digital TYP K thermometer with the sensor placed between the cuvette and the sample holder (GTH 1170 + GTF 300 GS, Greisinger, Regenstauf, Germany). Steady-state experiments were performed with an excitation slit with resulting band pass of 1 nm and an emission slit, which results in a band pass of 2–3 nm at an integration time of 1 s.

## 3. Results and Discussion

### 3.1. Analysis of Molecular Dynamics Calculations

**Figure 2:**
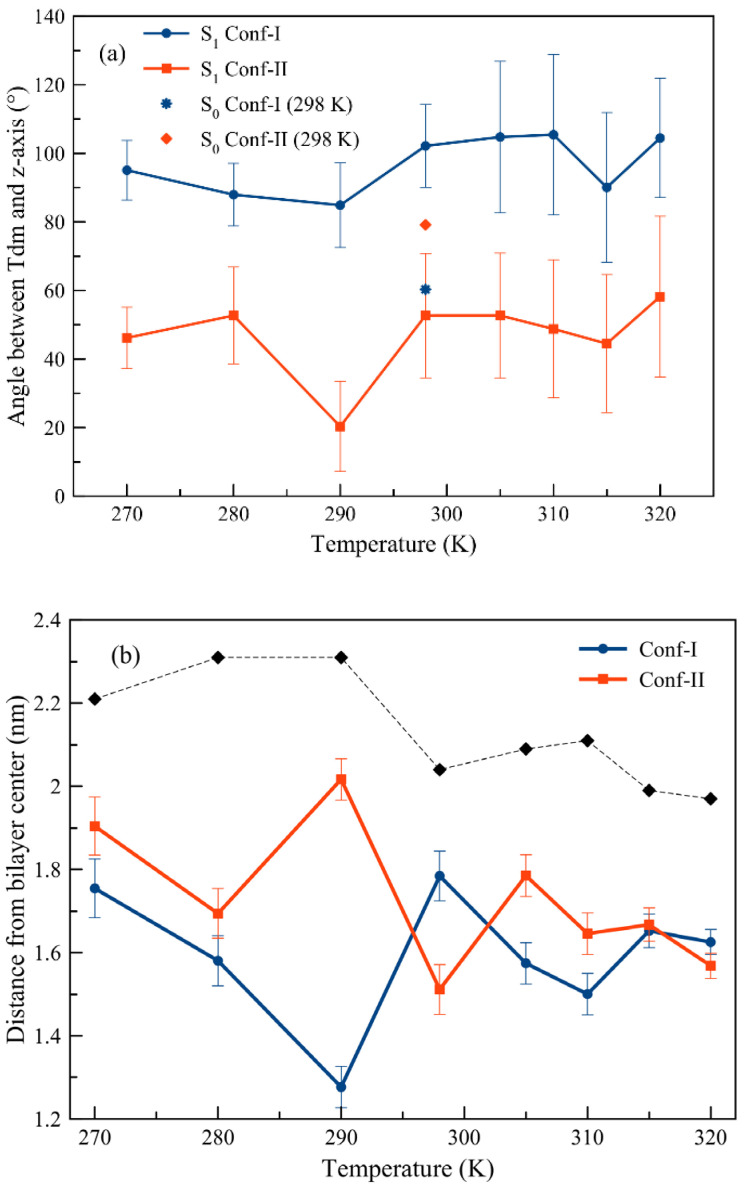
(**a**) The maximum of the distribution of the angles of the transition state dipole moment (‘tdm’) with respect to the *z*-axis of the DPPC membrane at different temperatures. The calculations were carried out using the S_1_ excited state for Conf-I and Conf-II. For 298K, the angle for the ground state of each conformer is given, too; (**b**) distance (nm) of the head group of Laurdan in the S_1_ excited state with respect to the center of the DPPC membrane (set at zero) at different temperatures. Black diamonds represent the averaged phosphorous atoms’ distance from the bilayer center, indicating the thickness of the DPPC membrane at different temperatures. The black, blue and red curves are typeset as a guide to the eye for phosphorous atoms, Conf-I and Conf-II, respectively. Error bars were computed considering HWHM of the distribution plots reported in Figures S2 and S8.

The obtained angles of the α-tilt (long molecular axis) and of the transition dipole moment (tdm) with respect to the membrane normal of both conformers of Laurdan in the S_1_ excited state embedded in the DPPC membrane (See Figure 2 and Figure S2 in Supplementary Information) can be compared with those obtained for the ground state (see Figure S3 or Figures 6 and S4 in Ref. [26]).

**Figure 3:**
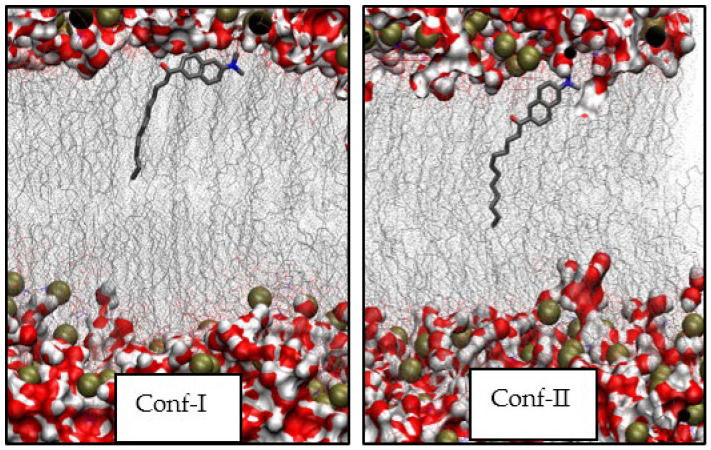
Representative depictions of electronically excited Conf-I and Conf-II in DPPC at 298K. Phosphor atoms are given in brown and oxygen and nitrogen ones in red and blue, respectively.

At temperatures as low as 270K and 280K, the angles between the tdm and the *z*-axis for Conf-I are at a minimum of 90° and 85°, respectively. This also holds true for a temperature of 290K. In comparison, for Conf-II, the angles for 270K and 280K do not substantially differ and amount to ~50°, while at 290K, this conformer is found to be rather parallel to the *z*-axis of the membrane as the angle shifts down to 30°. For Conf-I at 298K, the orientation of the head of Laurdan and its long axis changes from parallel (angle of ~40°) in the ground state to perpendicular (~80°) to the *z*-axis in the S_1_ excited state. The orientation of the tdm confirms this view as the angle changes from ~60° to ~100° for the ground and S_1_ excited states, respectively. For Conf-II, the orientation of the chromophore head changes from ~100° to ~70° upon excitation. For the tdm, a change from ~80° to ~55° was noted. This leads to a different shape of the conformers, as found in the electronic ground state. In fact, now Conf-I is found in a L-shape, while Conf-II is rather elongated. The characteristic positions are depicted in Figure 3. Figures S4 and S5 show that at all investigated temperatures, the amino group of the head of Conf-II is found further from the membrane center compared to the carbonyl one.

An increase in the temperature from 298K to 320K for Conf-I results only in a marginal enhancement in the angles. The orientation of Conf-I at 315K as this configuration seems to be less tilted with respect to the *z*-axis compared to the ones at 310K and 320K, although within the error of the method. We note that the transition temperature for DPPC is known to be 314.4K [51]. For Conf-II, the observed change in angles towards higher temperatures is analogous to what is observed for Conf-I. For Conf-II, from 310K, the angle of Laurdan’s head group with the *z*-axis increases steadily as the one for the tdm goes over 50° at 315K to 80° at 320K.

The optical excitation of the probe does not influence the flexibility of the molecule in the DPPC membrane; throughout the simulation window and for the considered range of temperatures, Conf-I and Conf-II do not interchange. This result counts, thus, for the excited Laurdan probe acting as a molecular rotor when embedded in a DPPC bilayer membrane in all the different liquid crystals, solid gels as well as in liquid disordered phases considered (see Figure S4).

In Figure 2b, the distance between the head group of Laurdan in the S_1_ excited state with respect to the center of the membrane is expressed as a function of the temperature (the complete densities are given in Figures S5–S7). For Conf-I, the head group moves towards the center of the membrane when the temperature is raised from 270K to 290K, while at 298K, the head group is found at the outer regions of the membrane. The effect of the orientation of both conformers can easily been seen, especially for 290K; the carbonyl oxygen in Conf-I is found closer to the membrane center compared to the one in Conf-II, where the abundance curves are broader compared to Conf-I (see Figure S8). At even higher temperatures, the head group moves again inwards. For Conf-II, the same shift towards the membrane center is observed for the lowest temperatures; however, the innermost position is now found for 298K. At higher temperatures, the headgroup of Laurdan moves towards the water layer. Conf-II stays largely in the same place in the membrane when the temperature is increased from 310K over the transition temperature to 320K. As a consequence, the difference between the positions of Laurdan is at its maximum at 290K. The environments of both conformers are intrinsically different, as Conf-I is located in the lipid tail region, while Conf-II is rather at the outside edge of the membrane, surrounded by an important amount of water molecules. Based on our studies of (non)linear optical properties of probe molecules in lipid bilayers and proteins, in which we discriminated between the influences of probe position, conformation and environment [23,27,29], we foresee now as well that the optical spectra of both conformers are significantly different. When experimentally oriented fluorescence studies of Laurdan in the solid gel phase are interpreted, it is, thus, of the utmost importance to take into account the differences in both position and orientation between the two conformers.

As a conclusion, the analysis of the orientation and position of the Laurdan probe indicate a particular character of the DPPC membrane phase at 290K and, to a lesser extent, at 298K. The orientation and position of Conf-II at this temperature differ profoundly from the ones at the other temperatures. On the other hand, the L_β’_ to L_α_ phase transition temperature around 315K mainly influences Conf-I.

### 3.2. Abundance of Water Molecules around Laurdan

A view on the radial distribution functions for water (Figure S9) illustrates the positional and orientational differences between both conformers. It can be seen that, while at a low temperature of 280K, Conf-I is surrounded by less water than Conf-II, as up to the second solvation shell, ~18% and ~30% of water molecules can be found, after the phase transition (320K), the inverse is observed. An analysis of hydrogen bonds in the second solvation shell (up to 5 Å from the Laurdan’s head) confirms this view. At low temperature, twice the amount of hydrogen bonds are found for Conf-II compared to Conf-I, while at higher temperature, the amount of hydrogen bonds for Conf-II decreases (see Table S1 and Figure S10). From these plots, the special nature of the DPPC lipid bilayer at 298K emerges; the radial distribution function describes values which are considerably lower than the ones obtained at other temperatures, especially for Conf-II (see Table S1). This decrease in value is related to the decreased distance from the centre of the membrane (see discussion below). The slope and the progression of the curve at larger distances is found to be different, too.

The change in the orientation of the water molecules in the neighborhood of the probe after the onset of excitation of Laurdan for both conformers is depicted in Figures S11 and S12. The solvent orientation is measured as <cos θ> with θ the angle between the vector pointing from the center of mass of the Laurdan head group to the oxygen atom of the water molecule, with the vector from this oxygen atom to the middle point between both H atoms of the same water molecule (see Figure S13). This means that a negative value is obtained when the two vectors have the same orientation, while a positive value is present when the orientation is opposite. For positive values, where the environment is non-equilibrated after excitation, the two hydrogen atoms point towards the Laurdan head, while for negative values, where the environment is equilibrated after excitation, the oxygen atom points towards the probe. A summary of the values is given in Table 1. For both conformers and at all simulated temperatures, reorganization of the water molecules is seen, as the cumulative solvent orientation after the first nanosecond differs from the one at later times, when the relaxation is reached. For Conf-I, the solvation shell after 3 ns has already adapted its orientation to the new electronic headgroup properties, which is considerably shorter than the probe’s excited state lifetime of 9 ns [27]. For Conf-II, which has a lifetime of 5 ns, the curves show a larger disturbance due to the excitation of the probe. At delay times up to 21 ns, the cumulative solvent orientation is generally negative. At only 6 ns after excitation, however, the obtained values strongly differ (see Table 1). At 305K, for instance, a value of −1.19 is obtained for 6–7 ns, compared to −3.57 for 21 ns. Based on our data, it can, thus, be said that the electronic configuration of Conf-II returns to the ground state before the environment fully adapts to the excited state configuration of the probe.

For Conf-I, the opposite trend is observed, since the relaxation of the environment is found to be quicker than the excited state decay. The cumulative solvent orientation curves are more regular for this conformer (see Figure S11). For 310K, at 0–1 ns, a value of −3.63 is obtained, compared to −4.68 at 20–21 ns. In between both time windows, the solvent orientation shifts from more negative to positive before it diminishes again. This difference in solvent behavior is also reflected in the different fluorescence response of the two conformers.

The explanation for this different behavior can be traced back to the equilibrium position of Laurdan in its ground state. From the radial distribution functions at room temperature, it follows that the first and second solvation shells for Conf-II contain less water, while this conformer is also located deeper in the membrane, according to our previous study [23]. Hence, it can be expected that Conf-II will interact differently with its surroundings when it is embedded in lipid bilayer membranes with lipids with a shorter fatty acid chain length than DPPC.

As a consequence, the through-space interaction between the neighboring, relatively densely packed water molecules is higher for Conf-I than for Conf-II in DPPC. The water environment of Conf-II reacts, therefore, noticeably slower than that of Conf-I. These relaxation times for the surrounding water molecules are in line with literature data. In pure solvent, the water reorganization takes place at timescales below 100 ps (see [52] and references therein), while Sykora et al. reported relaxation times up to 1.5 ns for Patman based on Time-Resolved Emission Spectroscopy (TRES) [53]. The authors state as well that the water relaxation times increase when the distance between the probe and the water layer increases, which is in full agreement with the findings of the current study. These observations are additionally enforced by the difference in membranes and membrane phases, as Sykora et al. based their research on probes embedded in a liquid disordered DOPC membrane rather than in a solid gel DPPC one, which is less prone to intruding water molecules. For water molecules isolated from the bulk and located around the probe, the membrane hinders the relaxation of their orientation.

### 3.3. Phase Changes of the Membrane

When the averaged area per lipid (APL) is considered (see Table S2), the different DPPC lipid phases can be identified along with the varying influence of Conf-I and Conf-II. An analysis of the deuterium order parameters permits one to visualize the phase changes in the lipid membrane and the varying interaction with the two conformers of Laurdan. Except for 290K and 305K, where the APLs for both conformers are very comparable, the membrane bilayers with Conf-II showcase higher values than the ones with Conf-I. This is the consequence of the differing orientation of the conformers, with Conf-I and Conf-II more parallel to the membrane surface and membrane tails, respectively. Conf-II, therefore, has a stronger interaction with the lipid tails than Conf-I. A strong increase in the APL is seen at the transition temperature towards the L_α_ phase. The analysis of the APL with Conf-I and Conf-II (see Table S2) clearly shows the appearance of the different phase transitions. The APL increases from ~51 Å^2^ for the L_c_ phase to 57 Å^2^ for L_α_. For L_β’_ and P_β’_, a value of ~52 Å^2^ was obtained through the simulations, which is in excellent agreement with the 52.3 Å^2^ value measured through X-ray diffraction at 298K [54,55].

**Figure 4:**
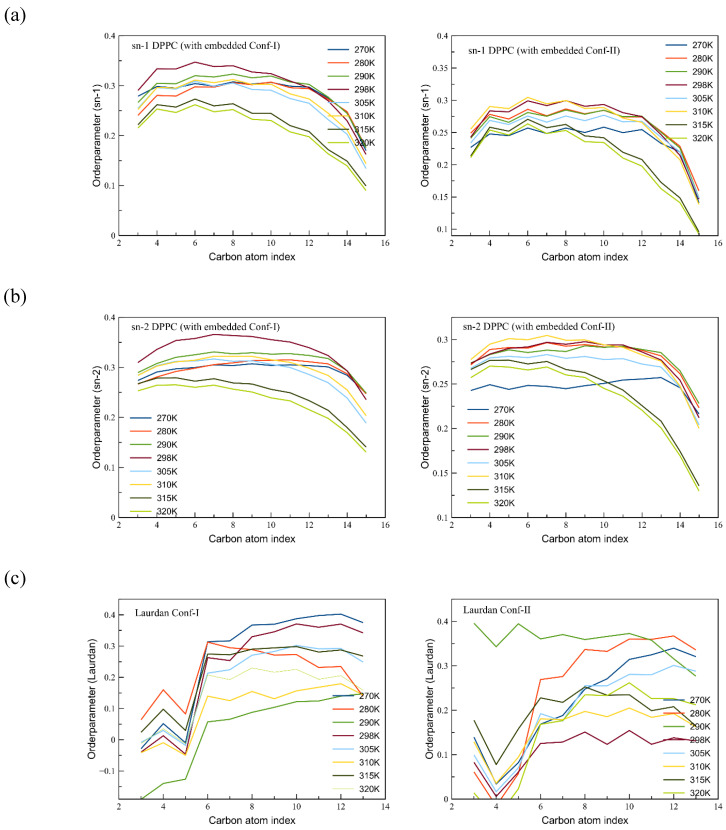
Order parameters for the (**a**) sn-1 and (**b**) sn-2 tails of DPPC. The ones for the tail of Laurdan are shown as well in (**c**). In the left (right) column, the tails are shown for the membrane in which Conf-I (Conf-II) is embedded.

In Figure S14, the APLs of the here-simulated lipid membranes with Conf-I and Conf-II are compared with the ones obtained by a comparable Gromos force field by Leekumjorn and Sum [56]. The APL trend going from L_β’_ towards the two phase transitions at higher temperatures is reproduced in the current study. On the other hand, the obtained values are underestimated, which is partly due to a lower cutoff of the Coulomb interaction. Although effects due to limited simulation time might play a role as well, it can be stated that the presence of Laurdan has a non-negligible effect on the membrane properties, which hampers a direct comparison with a pure DPPC membrane. As can be seen in Figure S15, the presence of Laurdan has a disrupting role in the local membrane environment for Conf-I due to its L-shape, while for Conf-II and its more elongated shape, the impact is minor. This difference is most striking at 298K, while at 320K, virtually no effect can be attributed to the different conformers as the obtained values are the same. The subphase at 298K also appears in the order parameter analysis (Figure 4). In particular, for Conf-I, the lipid tails at this temperature give the highest values. The L_β’_ and L_α_ phases can be identified as well in the different slopes of the curves. The L-shape for Laurdan is clearly seen in the lower values for the lower temperatures of the Conf-II tail. Due to the increased amount of water (compare, e.g., 280K for both conformers in Figure S9) in the neighborhood of Conf-II, the order of parameters is rather modest and points at a dynamic tail without a strong and fixed orientation.

**Figure 5:**
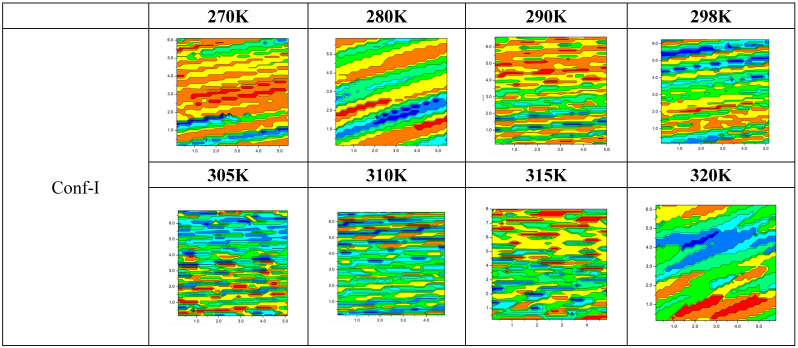

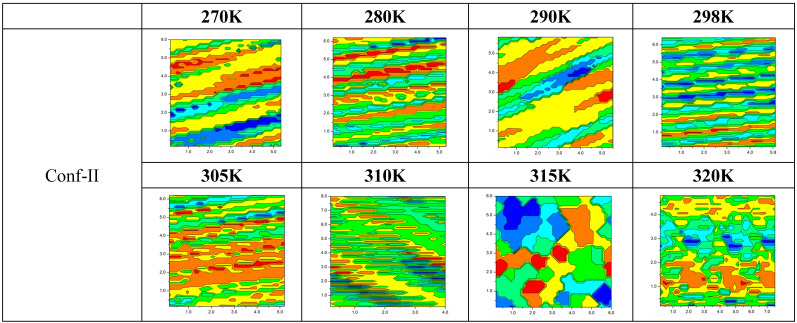
Two-dimensional thickness plots, showing the ripple of the membrane while increasing T and going through different phases along the x-y plane (box size considered) when the Laurdan conformers are present. The last frame of MD is considered as representative. Colors refer to the z direction (thickness), with blue the lowest (3.0 nm) and red the highest (5–5.4 nm) thickness, respectively.

In Figure 5, the last frames of the MD production run at different temperatures are considered, and their thicknesses are compared. For the L_c_ phases at 270K and 280K, large structures, valleys and peaks are seen at the surface and extend over the whole membrane. For the solid gel phases (290–305K), systematic patches are seen in these structures over the whole surface. The plots at 290K confirm the different interaction of both conformers with the environment; it is clear that Conf-II in a rather broad membrane has the possibility to be only slightly tilted with respect to the *z*-axis, while Conf-I in a less inflated membrane tends to be more parallel to the surface. For Conf-I, both the amino and carbonyl groups are located deeper in the membrane, while for Conf-II, the amino group interacts with the high-headgroup-density region, and the carbonyl oxygen is found closer to the membrane center.

At 298K, the patches break down and individual differences in thickness can be identified, while above 315K, global patterns at a considerable large scale appear (see Table S3). These depictions are in agreement with the reported order parameters. This later analysis proves once more that information about the position and orientation of Laurdan discloses important characteristics of the membrane phase. We would like to recall here that all calculations started from the same orientation and position of the probe. In all phases, the movement of the probe at the respective temperatures is different. Since we have shown that the probe affects the membrane, the orientation of this asymmetric probe matters and can help in defining the membrane phase. In addition, three different transitions are commonly reported between L_α_ and L_β’_ phases: sub-transition at 298K, pretransition at 306K and the main transition at 314K. The P_β’_ ripple phase can be found between pretransition and main transition, while below the pretransition temperature, a secondary ripple phase called L^d^_β_ was identified. Despite the limited size of the membrane considered in our computational protocol, and as can be seen in the depictions of Figure 5 and in Figure S16 with the lipid arrangements characteristic to the different ordered phases, we are able to observe all the mentioned transitions, thus validating the robustness of our methodological approach.

### 3.4. Optical Properties

**Figure 6:**
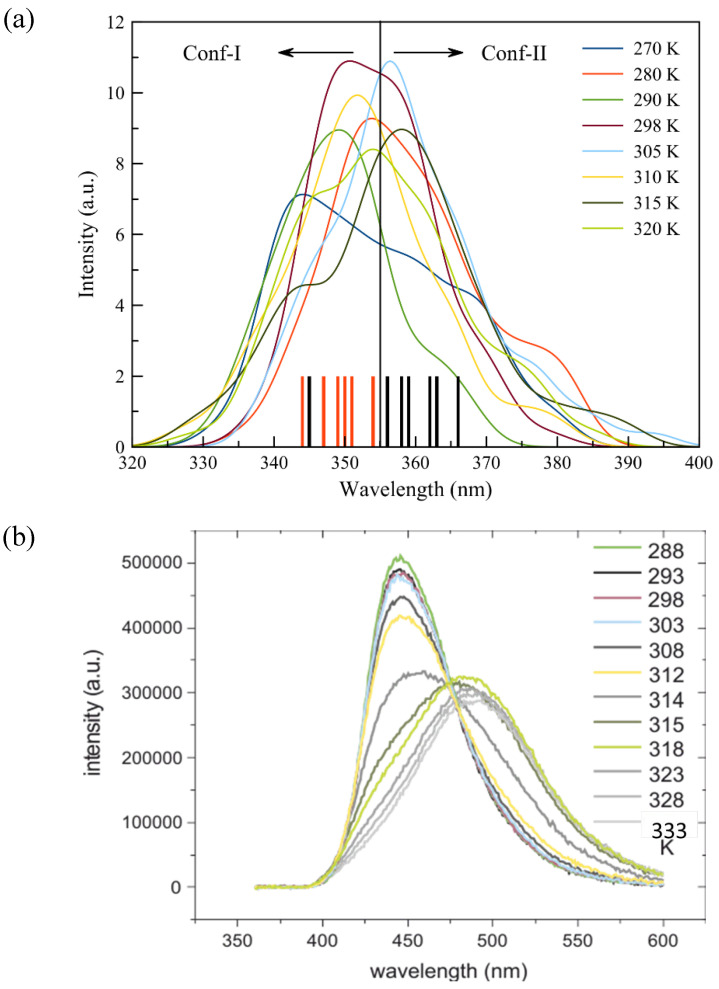
(**a**) Convoluted computed and (**b**) experimental fluorescence spectra of Laurdan embedded in DPPC at different temperatures. The vertical bars in (**a**) denote the positions of the maximal peaks at the different temperatures.

The obtained fluorescence spectra depicting the summed emission from the S_1_ excited states of both conformers are reported in Figure 6. When the temperature is raised from 270K to 315K and the fluorescence of Laurdan is investigated regardless of the conformational differences, a red shift of ~16 nm is obtained. From 290K to 315K or from the L_β’_ to the L_α_ phase, this shift amounts to ~11 nm.

In our theoretical results, lower-intensity shoulders in the 375–400 nm window with a ~15 nm longer wavelength with respect to the maximum of the peak are seen for all temperatures. These results, albeit blue shifted, are in agreement with experimental fluorescence spectra (Figure 6b), in which red-shifted shoulders of ~15–20 nm are reported for the 288–312K (15–39 °C) temperature range. The experimental data show as well that the shoulders disappear at 315K (42 °C), for which a larger, symmetric density profile is indeed found. At the main phase transition between 314K and 315K, the emission changes abruptly: the experimental spectra report a broad and rather asymmetric profile with a maximum at ~450 nm and a longer tail at 314K (41 °C), while at 315K (42 °C), the peak shifts towards 490 nm.

A deconvolution of Figure 6 for both conformers along with a discussion and an analysis of the excited state (Table S4) is given in Figures S17 and S18. As can be seen from a comparison with Figure 6, the main peak of the curve is related to the contribution of Conf-I, while the reported shoulders in the overall spectrum coincide with the maximum of the fluorescence curves for Conf-II. It can be seen that the fluctuations for the averaged emission wavelengths of Conf-I reach ~19 nm, while the range for Conf-II spans ~26 nm. Moreover, from the comparison between our simulated and experimental fluorescence spectra, it is clear that differences below 10 nm in fluorescence maxima for both conformers are very challenging to experimentally unravel, especially for the L_α_ phase. As has been argued previously, the bilayer system at 290K extends the current discussion, as Conf-II has a fluorescence peak at a wavelength of 345 nm, which is shorter than the wavelengths obtained at other temperatures (Figure 6). In addition, from computation, we can conclude that the maximal values for Conf-I exhibit a global red shift from low temperatures to higher ones, which is related to a softening of the tissue and an increase in the diffusivity of the membrane and a protruding effect of water. On the other hand, Conf-II is less affected by the environmental changes due to the increased temperature. This difference in the response of the two conformers is highlighted when the ripple phase is considered (at 305K). At this temperature, the fluorescence spectrum of Conf-I is clearly different than at other temperatures, but for Conf-II, the presence of a ripple phase does not affect the optical properties. The different impacts of the environment on the two conformers become relevant when the fluorescence decay time is considered.

**Figure 7:**
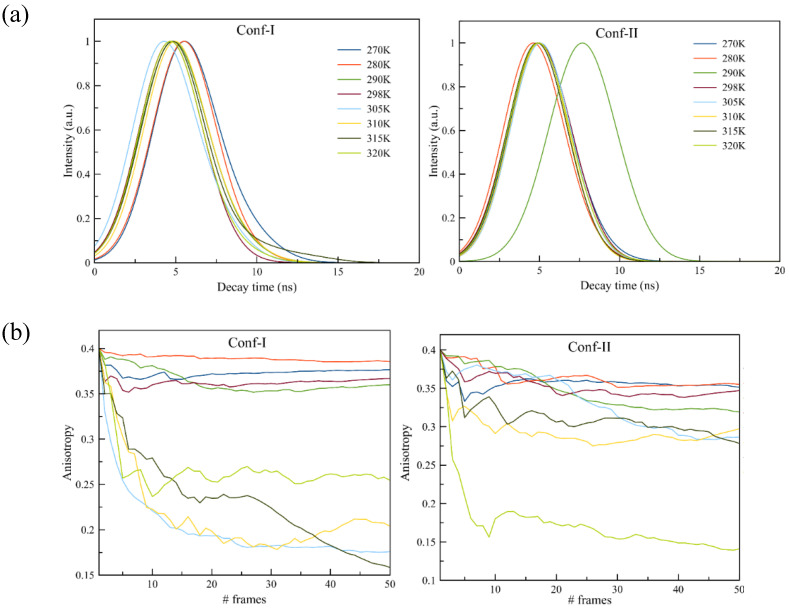
(**a**) Fluorescence decay time computed for Laurdan embedded in DPPC at different temperatures given as normalized histograms for both conformers; (**b**) fluorescence anisotropy for Laurdan embedded in DPPC at different temperatures for both conformers.

To disentangle the conformational effect from the presence of different phases, we performed additional fluorescence analyses, namely the decay time and the anisotropy decay. From the fluorescence decay time analysis reported in Figure 7, a clear trend is present for Conf-I; increasing the temperature, the decay time decreases towards lower values, going from 6 ns to 4.9 ns. Clearly, for this conformer, the environment has a strong effect on the optical properties, due to its orientation, position and conformation. On the other hand, Conf-II shows little variation while increasing the membrane temperature, with decay time values in the 4.7–5.1 ns range, once more suggesting the weak effect of the surroundings over the fluorescence properties of this conformer. Once more, an outlier is present at 29K, with a very high decay time of 7.7 ns. As mentioned earlier, Conf-II in the DPPC membrane at 290K is oriented almost parallel to the *z*-axis and located at the interface between the membrane head and water (Figure 2). We remind the reader here that this temperature is at the onset of the solid gel phase.

Experimentally, the lifetimes of Laurdan were obtained for both channels at 440 nm and 490 nm (see Table 2 and decay curves in Figure S19). Confirming the theoretical data, for both channels, the lifetime decreases with increasing temperature. We find that the lifetime values for Conf-I can be used to identify the membrane phase. Summarizing our results, the crystal phase L_c_ bears the longest lifetime, followed by the gel phase at 290K (17 °C) and finally the liquid disordered one, for which the shortest time is found. The rippled phase P_β’_ observed at around 310K (37 °C) in our computational data has a slightly longer lifetime than in the gel phase. In the experiments, we did not finetune the temperature to pinpoint the phase differences for which fingerprints can be obtained from the computational analysis behind the temperatures of 298K and 305K.

The same trend already shown is also present in the simulations of the fluorescence anisotropy decay, which clearly shows the effect of the membrane on Conf-I and its negligible contribution for Conf-II (see Figure 7b). In detail, for Conf-I, when the membrane is in its L_c_ state, the anisotropy is kept constant to a value close to 0.38. For 290K and 298K, the obtained value of 0.36 is only marginally lower. At 305K and 310K, it exhibits the strongest decay curves. Once the transition temperature towards the L_α_ phase is reached, the curves are steep and seem to converge at values around 0.25. We note here the loss of correlation at higher frame numbers (above #30) for the depicted curve of 315K. The same analysis for Conf-II shows very different results. Now, much stronger anisotropy is present (with values between 0.35 and 0.29) up to 315K, well into the L_α_ phase. Only at a higher temperature (320K) is the expected decay in anisotropy observed. This is a direct consequence of the remarkable broad distribution of the orientational distribution function of Conf-II (see Figure 2), and it is due to the changing ripples of the membrane at this temperature (as discussed above).

**Figure 8:**
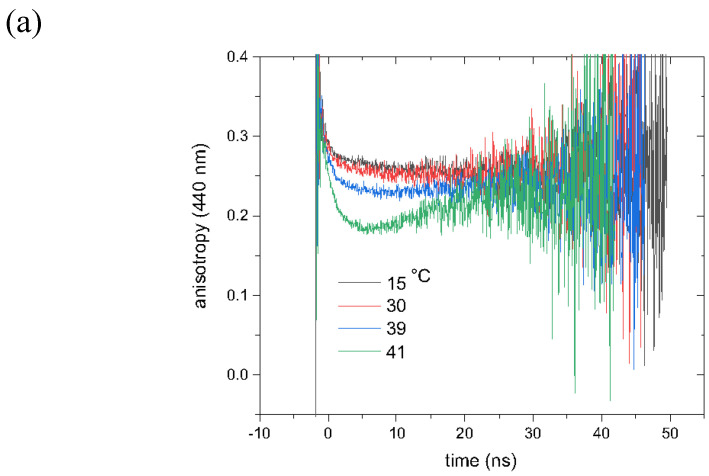

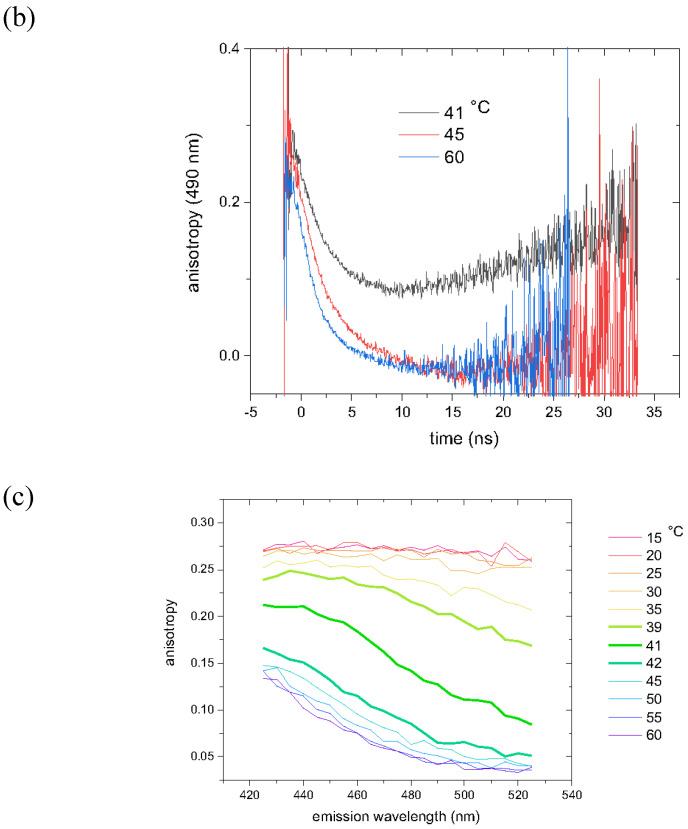
Experimental fluorescence anisotropy data of Lauran embedded in a DPPC membrane. In (**a**), the results for the 440 nm channel are used, while in (**b**), the one for the 490 nm is plotted. In (**c**), the experimentally obtained steady-state fluorescence decay curves are given as a function of emission wavelength. Also, 1 mM laurdan embedded in DMP has been used along with 10 mM Tris pH 7 and 100 mM NaCl.

These results were once more validated by experimentally oriented time-dependent anisotropy studies (Figure 8), in which the influence of both conformers can be clearly observed. At 41 °C and in P_β’_, a turn-up in the signal is present, which can only be attributed to the presence of two, or, in principle, more, components. At higher temperatures, the anisotropy generally decays again, while the slope even increases towards 60 °C. The monotonous decaying curves at higher temperatures point at a liquid disordered phase, in which both conformers of Laurdan interchange easily. The slope increases for both channels as the temperature rises, while the time constant of the probe at 440 nm steadily decreases from 3.61 ns at 15 °C in the gel phase to 1.39 ns at 41 °C, and at 490 nm from 2.70 ns in the P_β’_ to 2.41 ns in the L_α_ phase. In the latter case, at 45 °C and 60 °C, the decay is found to be complete. The decay time constants and the relative abundance for both conformers are further reported in Table S5.

The steady-state anisotropy studies showcase the ripple phase P_β’_, too. In Figure 8, the isolated case for 314K (41 °C) can be clearly distinguished from the closely packed anisotropy lines in the solid gel phase at 290K (15 °C) or at the ones of Laurdan embedded in a liquid disordered phase above 315K (42 °C). The steady-state results confirm once more the possible use of the probe to identify different environments. As we clearly demonstrated throughout the study, this is largely due to the position and orientation of Conf-I, which determine its interaction with its surroundings. As we assign the “odd” behavior of the time-resolved anisotropy at 41 °C/440 nm to two populations with different lifetimes, we fitted the time-resolved fluorescence with two components (see Table 2). For 440 nm, it is visible from the decay lines that this is not a purely mono-exponential decay. On the other hand, 490 nm can only be fitted with a mono-exponential curve. To prove the correctness of these results, we verified that the fitted lifetimes allow for the simulation of the time-resolved anisotropy at 41 °C/440 nm (Figure S20). From these fitting values, it seems that below the phase transition, ~20% of the fast population persists, whereas the fast population dominates the system above the phase transition. This is an additional confirmation of the presence of either conformer of Laurdan in the DPPC membrane at different temperatures.

## 4. Conclusions

The positions and orientations of optically excited conformers of Laurdan are investigated in a DPPC membrane at various temperatures. They differ through the orientation of the carbonyl oxygen, which points either toward the β-position of the naphthalene core (Conf-I) or to the α-position (Conf-II). Analogously to the ground state, the conformers do not interchange in this membrane, for the entire temperature range considered (270–320K). However, the position of the two conformers in the membrane changes differently with temperature. Due to a varying interaction with neighboring water molecules in combination with the different excited state lifetimes of the two conformers, the environment relaxation times differ, too. However, for Conf-I, the rather intuitive case is observed as the excited state lifetime is longer than the relaxation of the environment, but for Conf-II, the environment is not yet adapted when the probe returns to the ground state. At the transition from L_c_ to the L_β’_ phase at 290K, the differences between the position, orientation and the properties of the two conformers are the strongest. The difference in simulated decay time at this temperature between both conformers amounts to 4 ns. The time-dependent experimental fluorescence anisotropy data confirm the presence of two conformers, which behave differently at different temperatures. Throughout the temperature range, the different membrane phases can be identified through the lifetime and anisotropy decay curves.

In this work, we assessed the influence of the orientation of Laurdan’s carbonyl group and its tail on the optical properties and the consequences for fluorescence experiments. Back in 1997, Parassassi et al. investigated GP profiles of Laurdan for phospholipid vesicles. The authors concluded that the observed change in the apparent GP value was related to a change in local orientation and that different orientations selected different environments [30]: “There must be an intrinsic GP heterogeneity, either at the submicroscopic level (in this case the regions of different GP may simply correspond to regions of different lipid orientation) or in regions resolvable by the microscope (in which case the GP domains correspond to real ‘fluidity’ domains)”. In the current work, however, we state the importance of both conformers for the interpretation of such data. We point at the consequence of conformational differences on the optical properties in a membrane environment, while, in contrast to what was assumed previously, the lipid orientation over the different measurements stays the same.

The current work aims to clarify the properties of Laurdan for one specific lipid bilayer, which were not known before, and paves the way to treat the here-chosen molecular probe in more complex systems. In view of the wide use of the probe in fluorescence experiments, we hope that this work on Laurdan will contribute to an increased understanding and a profound screening of cell membranes and tissues, which are vulnerable to cancerous or inflammatory transformations.

## Figures and Tables

**Table 1:** Cumulative solvent orientation at different time delays after excitation, obtained at 1 nm from the mass center of the Laurdan head group for the two conformers of Laurdan in the DPPC membrane *^a^*.

	Conf	0–1 ns	3–4 ns	6–7 ns	9–10 ns	12–13 ns	20–21 ns
270K	I	−3.22	−1.62	−4.88	−6.06	−6.43	−2.76
	II	−1.93	−3.96	−4.99	−8.36	−4.57	−3.36
280K	I	−0.19	−5.71	−1.01	−0.42	−1.77	−2.68
	II	−1.15	−5.84	−1.45	−6.14	0.16	−5.20
290K	I	−4.03	0.53	0.58	−1.89	1.96	0.10
	II	0.62	−0.24	−3.17	−6.19	−2.51	−1.35
298K	I	−3.03	−6.27	−6.11	−4.71	−5.93	−7.59
	II	−1.22	1.56	−1.29	−3.15	−4.32	−5.43
305K	I	−3.59	−2.30	-6.70	−2.45	−3.25	−6.28
	II	0.17	−0.24	−1.19	−2.80	−5.11	−3.57
310K	I	−3.63	−5.19	−8.55	−1.52	1.00	−4.68
	II	−2.25	−6.23	−3.22	−6.75	−1.88	−3.97
315K	I	−3.41	−3.85	−3.64	−7.49	−3.18	−1.01
	II	−1.35	−0.45	−0.63	1.89	−2.22	−4.09
320K	I	−3.59	−7.66	−0.51	−1.11	−2.67	−2.94
	II	−2.46	−3.07	−5.19	−1.20	−5.35	−1.66

*^a^* The values obtained for other distances can be obtained from the plots given in Figures S10 and S11.

**Table 2:** Experimentally obtained lifetimes for Laurdan obtained at the channels at 440 nm and 490 nm *^a^*.

	440 nm Slow Comp.	440 nm Fast Comp.	490 nm
15 °C	7.70 ± 0.23 (73 ± 10)%	5.04 ± 0.58 (27 ± 10)%	
30 °C	7.31 ± 0.12 (82 ± 4)%	3.98 ± 0.49 (18 ± 4)%	
39 °C	7.15 ± 0.08 (78 ± 2)%	3.28 ± 0.21 (22 ± 2)%	6.67 ± 0.03
41 °C	7.46 ± 0.17 (43 ± 3)%	3.58 ± 0.14 (57 ± 3)%	5.40 ± 0.02
45 °C	7.68 ± 1.26 (4 ± 2)%	2.67 ± 0.05 (96 ± 2)%	4.10 ± 0.03
60 °C			3.32 ± 0.02

*^a^* Values given in ns and fractions in integrated intensities.

## Data Availability

Data available upon request to the corresponding authors.

## Supplementary Materials

The following supporting information can be downloaded at: https://www.mdpi.com/article/10.3390/cells13151232/s1, Figure S1: angle of the Laurdan TDM with the z-axis; Figure S2: Distributions of the angles of the transition state dipole moment; Figure S3: Comparison between the orientations of the tdm with respect to the z-axis; Figure S4: Distribution of the position of the N- and O-atoms in the headgroup of Laurdan excited in the S_1_ state; Figure S5: Distribution of the DPPC membrane at different temperatures; Figure S6: Distance of the density maxima of the N- and O-atoms in the head group of Laurdan; Figure S7: Distribution of the dihedral angles of Laurdan; Figure S8: Density distribution of the head group of Laurdan; Figure S9: Radial distribution functions of water; Figure S10: Hydrogen bonds; Figures S11 and S12: Cumulative solvent orientation; Figure S13: Depiction of the angle θ; Figure S14: Comparison between the APLs for the membranes; Figure S15: Representative snapshots of the position of the two conformers of Laurdan; Figure S16: Representative snapshots of the DPPC membrane; Figure S17: Fluorescence spectra of Laurdan; Figure S18: Evolution of the max and averaged emission; Figure S19: Experimentally obtained emission decay curves; Figure S20: simulation of the time resolved anisotropy; Table S1: Number of hydrogen bonds; Table S2: Averaged area per DPPC lipid; Table S3: Averaged thickness of the membrane; Table S4: Detailed analysis of the transitions; Table S5: Fitted time constants and coefficients for the anistropy decay.

## References

[1] Tenchov B., Koynova R., Rapp G. (2001). New Ordered Metastable Phases between the Gel and Subgel Phases in Hydrated Phospholipids. Biophys. J..

[2] Walter V., Ruscher C., Gola A., Marques C.M., Benzerara O., Thalmann F. (2021). Ripple-like instability in the simulated gel phase of finite size phosphocholine bilayers. Biochim. Biophys. Acta (BBA)—Biomembr..

[3] Nagarajan S., Schuler E.E., Ma K., Kindt J.T., Dyer R.B. (2012). Dynamics of the Gel to Fluid Phase Transformation in Unilamellar DPPC Vesicles. J. Phys. Chem. B.

[4] Mills J.K., Needham D. (2005). Lysolipid incorporation in dipalmitoylphosphatidylcholine bilayer membranes enhances the ion permeability and drug release rates at the membrane phase transition. Biochim. Biophys. Acta (BBA)—Biomembr..

[5] Needham D., Dewhirst M.W. (2001). The development and testing of a new temperature-sensitive drug delivery system for the treatment of solid tumors. Adv. Drug Deliv. Rev..

[6] Haidekker M.A., Ling T.T., Anglo M., Stevens H.Y., Frangos J.A., Theodorakis E.A. (2001). New fluorescent probes for the measurement of cell membrane viscosity. Chem. Biol..

[7] Kuimova M.K. (2012). Mapping viscosity in cells using molecular rotors. Phys. Chem. Chem. Phys..

[8] Nagle J.F., Tristram-Nagle S. (2000). Structure of lipid bilayers. Biochim. Biophys. Acta (BBA)—Biomembr..

[9] Marsh D. (2013). Handbook of Lipid Bilayers.

[10] Axelrod D., Koppel D., Schlessinger J., Elson E., Webb W. (1976). Mobility Measurement by Analysis of Fluorescence Photobleaching Recovery Kinetics. Biophys. J..

[11] Dayel M.J., Hom E.F.Y., Verkman A.S. (1999). Diffusion of green fluorescent protein in the aqueous-phase lumen of endoplasmic reticulum. Biophys. J..

[12] Hendrix J., Dekens T., Schrimpf W., Lamb D.C. (2016). Arbitrary-Region Raster Image Correlation Spectroscopy. Biophys. J..

[13] Schrimpf W., Lemmens V., Smisdom N., Ameloot M., Lamb D.C., Hendrix J. (2018). Crosstalk-free multicolor RICS using spectral weighting. Methods.

[14] Kitamura A., Tornmalm J., Demirbay B., Piguet J., Kinjo M., Widengren J. (2023). Trans-cis isomerization kinetics of cyanine dyes reports on the folding states of exogeneous RNA G-quadruplexes in live cells. Nucleic Acids Res..

[15] Manzo C., Garcia-Parajo M.F. (2015). A review of progress in single particle tracking: From methods to biophysical insights. Rep. Prog. Phys..

[16] Xu H., Chmyrov V., Widengren J., Brismar H., Fu Y. (2015). Mechanisms of fluorescence decays of colloidal CdSe-CdS/ZnS quantum dots unraveled by time-resolved fluorescence measurement. Phys. Chem. Chem. Phys..

[17] Persson G., Thyberg P., Widengren J. (2008). Modulated fluorescence correlation spectroscopy with complete time range information. Biophys. J..

[18] Ameloot M., vandeVen M., Acuña A.U., Valeur B. (2013). Fluorescence anisotropy measurements in solution: Methods and reference materials (IUPAC Technical Report). Pure Appl. Chem..

[19] Osella S., Paloncyova M., Sahi M., Knippenberg S. (2020). Influence of Membrane Phase on the Optical Properties of DPH. Molecules.

[20] Osella S., Granucci G., Persico M., Knippenberg S. (2023). Dual photoisomerization mechanism of azobenzene embedded in a lipid membrane. J. Mater. Chem. B.

[21] Frank J.A., Franquelim H.G., Schwille P., Trauner D. (2016). Optical Control of Lipid Rafts with Photoswitchable Ceramides. J. Am. Chem. Soc..

[22] Klymchenko A.S., Kreder R. (2014). Fluorescent Probes for Lipid Rafts: From Model Membranes to Living Cells. Chem. Biol..

[23] Osella S., Smisdom N., Ameloot M., Knippenberg S. (2019). Conformational Changes as Driving Force for Phase Recognition: The Case of Laurdan. Langmuir.

[24] Bagatolli L.A., Mély Y., Duportail G. (2012). Fluorescent Methods to Study Biological Membranes.

[25] Parasassi T., De Stasio G., Ravagnan G., Rusch R.M., Gratton E. (1991). Quantitation of lipid phases in phospholipid vesicles by the generalized polarization of Laurdan fluorescence. Biophys. J..

[26] Parasassi T., De Stasio G., d’Ubaldo A., Gratton E. (1990). Phase fluctuation in phospholipid membranes revealed by Laurdan fluorescence. Biophys. J..

[27] Osella S., Knippenberg S. (2019). Laurdan as a Molecular Rotor in Biological Environments. ACS Appl. Bio. Mater..

[28] Hornum M., Kongsted J., Reinholdt P. (2021). Computational and photophysical characterization of a Laurdan malononitrile derivative. Phys. Chem. Chem. Phys..

[29] Osella S., Murugan N.A., Jena N.K., Knippenberg S. (2016). Investigation into Biological Environments through (Non)linear Optics: A Multiscale Study of Laurdan Derivatives. J. Chem. Theory Comput..

[30] Parasassi T., Gratton E., Yu W.M., Wilson P., Levi M. (1997). Two-photon fluorescence microscopy of Laurdan generalized polarization domains in model and natural membranes. Biophys. J..

[31] Bagatolli L.A., Gratton E., Fidelio G.D. (1998). Water dynamics in glycosphingolipid aggregates studied by LAURDAN fluorescence. Biophys. J..

[32] Bagatolli L.A., Parasassi T., Fidelio G.D., Gratton E. (1999). A model for the interaction of 6-lauroyl-2-(N,N-dimethylamino)naphthalene with lipid environments: Implications for spectral properties. Photochem. Photobiol..

[33] Parasassi T., Krasnowska E.K., Bagatolli L., Gratton E. (1998). Laurdan and Prodan as Polarity-Sensitive Fluorescent Membrane Probes. J. Fluoresc..

[34] Van Der Spoel D., Lindahl E., Hess B., Groenhof G., Mark A.E., Berendsen H.J. (2005). GROMACS: Fast, flexible, and free. J. Comput. Chem..

[35] Braun A.R., Sachs J.N., Nagle J.F. (2013). Comparing Simulations of Lipid Bilayers to Scattering Data: The GROMOS 43A1-S3 Force Field. J. Phys. Chem. B.

[36] Marrink S.J., Corradi V., Souza P.C.T., Ingólfsson H.I., Tieleman D.P., Sansom M.S.P. (2019). Computational Modeling of Realistic Cell Membranes. Chem. Rev..

[37] Chiu S.-W., Pandit S.A., Scott H.L., Jakobsson E. (2009). An Improved United Atom Force Field for Simulation of Mixed Lipid Bilayers. J. Phys. Chem. B.

[38] Gov N.S., Gopinathan A. (2006). Dynamics of Membranes Driven by Actin Polymerization. Biophys. J..

[39] Kukol A. (2009). Lipid Models for United-Atom Molecular Dynamics Simulations of Proteins. J. Chem. Theory Comput..

[40] Reif M.M., Hünenberger P.H., Oostenbrink C. (2012). New Interaction Parameters for Charged Amino Acid Side Chains in the GROMOS Force Field. J. Chem. Theory Comput..

[41] Frisch M.J., Trucks G.W., Schlegel H.B., Scuseria G.E., Robb M.A., Cheeseman J.R., Scalmani G., Barone V., Mennucci B., Petersson G.A. (2016). Gaussian 09 Revision B.01.

[42] Yanai T., Tew D.P., Handy N.C. (2004). A new hybrid exchange-correlation functional using the Coulomb-attenuating method (CAM-B3LYP). Chem. Phys. Lett..

[43] Berger O., Edholm O., Jahnig F. (1997). Molecular dynamics simulations of a fluid bilayer of dipalmitoylphosphatidylcholine at full hydration, constant pressure, and constant temperature. Biophys. J..

[44] Hess B., Bekker H., Berendsen H.J.C., Fraaije J. (1997). LINCS: A linear constraint solver for molecular simulations. J. Comput. Chem..

[45] Nose S. (1984). A Unified Formulation of the Constant Temperature Molecular-Dynamics Methods. J. Chem. Phys..

[46] Hoover W.G. (1985). Canonical dynamics: Equilibrium phase-space distributions. Phys. Rev. A.

[47] Osella S., Marczak M., Murugan N.A., Knippenberg S. (2022). Exhibiting environment sensitive optical properties through multiscale modelling: A study of photoactivatable probes. J. Photochem. Photobiol. A Chem..

[48] Aidas K., Angeli C., Bak K.L., Bakken V., Bast R., Boman L., Christiansen O., Cimiraglia R., Coriani S., Dahle P. (2014). The Dalton quantum chemistry program system. Wiley Interdiscip. Rev.-Comput. Mol. Sci..

[49] Dunning T. (1989). Gaussian-Basis Sets for Use in Correlated Molecular Calculations 1. the Atoms Boron Through Neon and Hydrogen. J. Chem. Phys..

[50] Grabarz A.M., Ośmiałowski B. (2021). Benchmarking Density Functional Approximations for Excited-State Properties of Fluorescent Dyes. Molecules.

[51] Buehler L.K. (2016). Cell Membranes.

[52] Lakowicz (2007). Principles of Fluorescence Spectroscopy.

[53] Sykora J., Jurkiewicz P., Epand R.M., Kraayenhof R., Langner M., Hof M. (2005). Influence of the curvature on the water structure in the headgroup region of phospholipid bilayer studied by the solvent relaxation technique. Chem. Phys. Lipids.

[54] Hartkamp R., Moore T.C., Iacovella C.R., Thompson M.A., Bulsara P.A., Moore D.J., McCabe C. (2016). Investigating the Structure of Multicomponent Gel-Phase Lipid Bilayers. Biophys. J..

[55] Katsaras J., Yang D.S., Epand R.M. (1992). Fatty-acid chain tilt angles and directions in dipalmitoyl phosphatidylcholine bilayers. Biophys. J..

[56] Leekumjorn S., Sum A.K. (2007). Molecular studies of the gel to liquid-crystalline phase transition for fully hydrated DPPC and DPPE bilayers. Biochim. Biophys. Acta.

