# Supplementary material for: Hydration- and Temperature-Dependent Fluorescence Spectra of Laurdan Conformers in a DPPC Membrane
Source: Cells. 2024 Jul 23;13(15):1232. doi: 10.3390/cells13151232 (PMC11311969; doi:10.3390/cells13151232)
Supplement: Supplementary file 1 [file cells-13-01232-s001.zip › cells-3093226-supplementary.pdf]

Supplementary information for

Hydration-temperature dependent fluorescence spectra of Laurdan conformers in a DPPC membrane.

S. Knippenberg,[1*] K. De,[2] C. Aisenbrey,[2] B. Bechinger,[2,3] S. Osella,[4,*]

[1] Hasselt University, Theory Lab, Agoralaan Building D, 3590 Diepenbeek, Belgium.

[2] University of Strasbourg/CNRS, UMR7177, Institut de Chimie de Strasbourg, Strasbourg, France.

[3] Institut Universitaire de France (IUF), Paris, France.

[4] Chemical and Biological Systems Simulation Lab, Centre of New Technologies, University of Warsaw, Banacha 2C, 02-097 Warsaw, Poland.

Email addresses: stefan.knippenberg@uhasselt.be (SK); s.osella@cent.uw.edu.pl (SO)

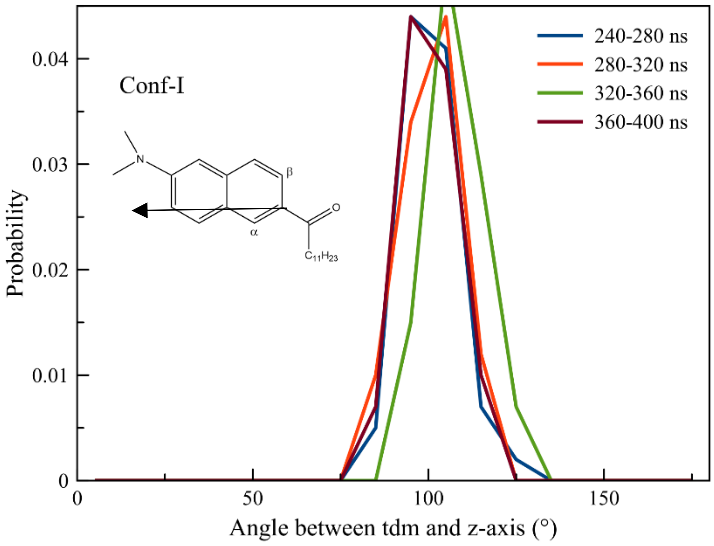

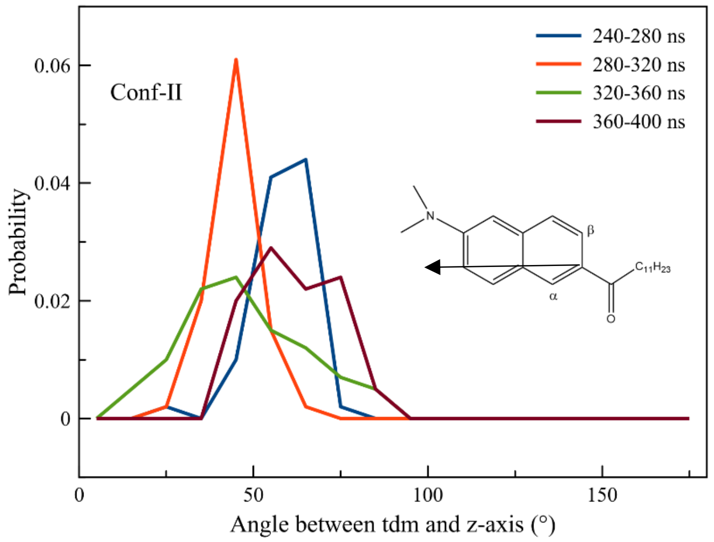

Figure S1 Variation of the angle of the Laurdan TDM with the z-axis for windows of 40 ns between 240 and 400 ns in both MD runs for 298K. The arrow in the inset indicate the orientation of the transition dipole moment vector considered for the analysis.

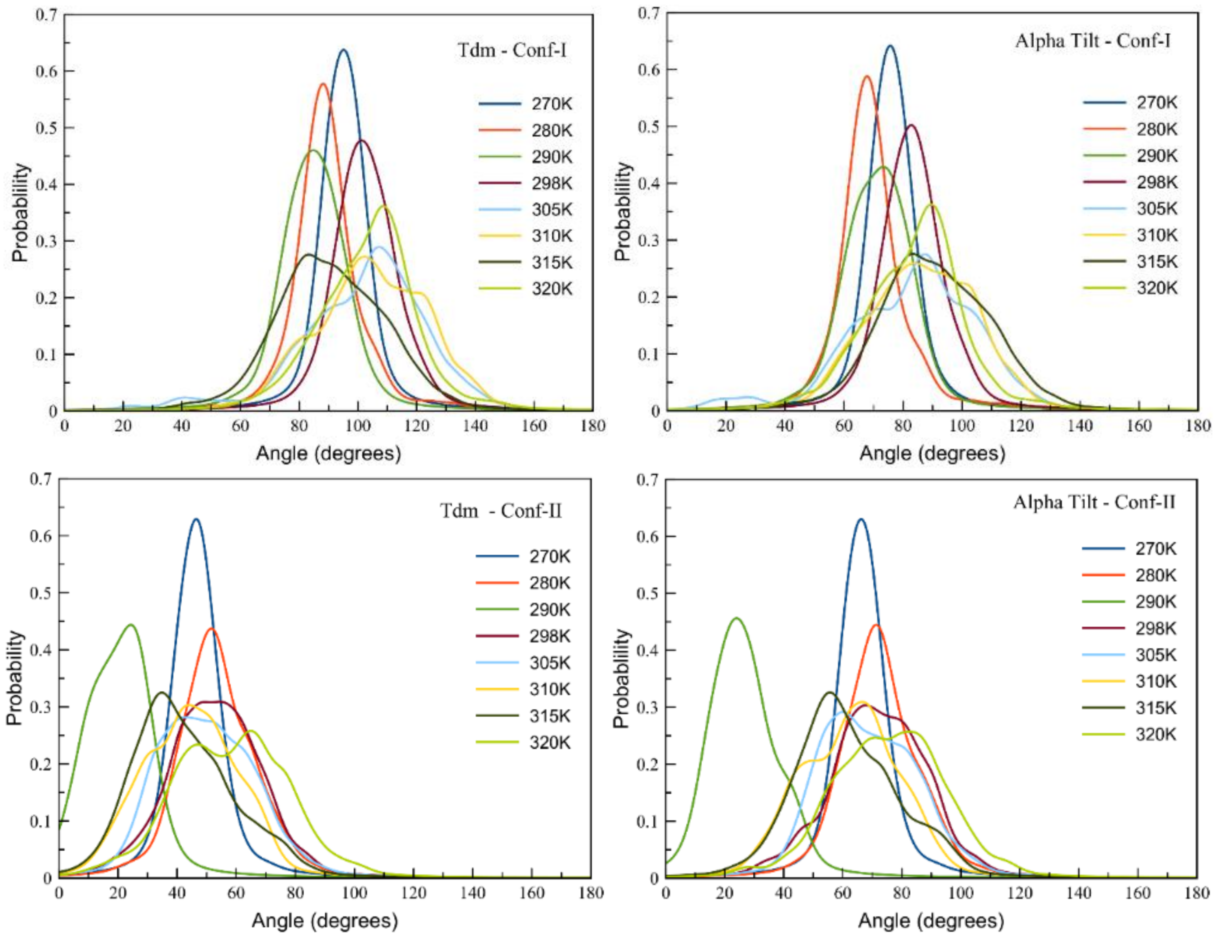

Figure S2 Distributions of the angles of the transition state dipole moment ('tdm', at the left hand side) as well as the long molecular axis ('alpha tilt', at the right hand side) with respect to the z-axis of the DPPC ($L_{\beta'}$) membrane at different temperatures. The calculations have been done using the S1 excited state for Conf-I (top) and Conf-II (bottom).

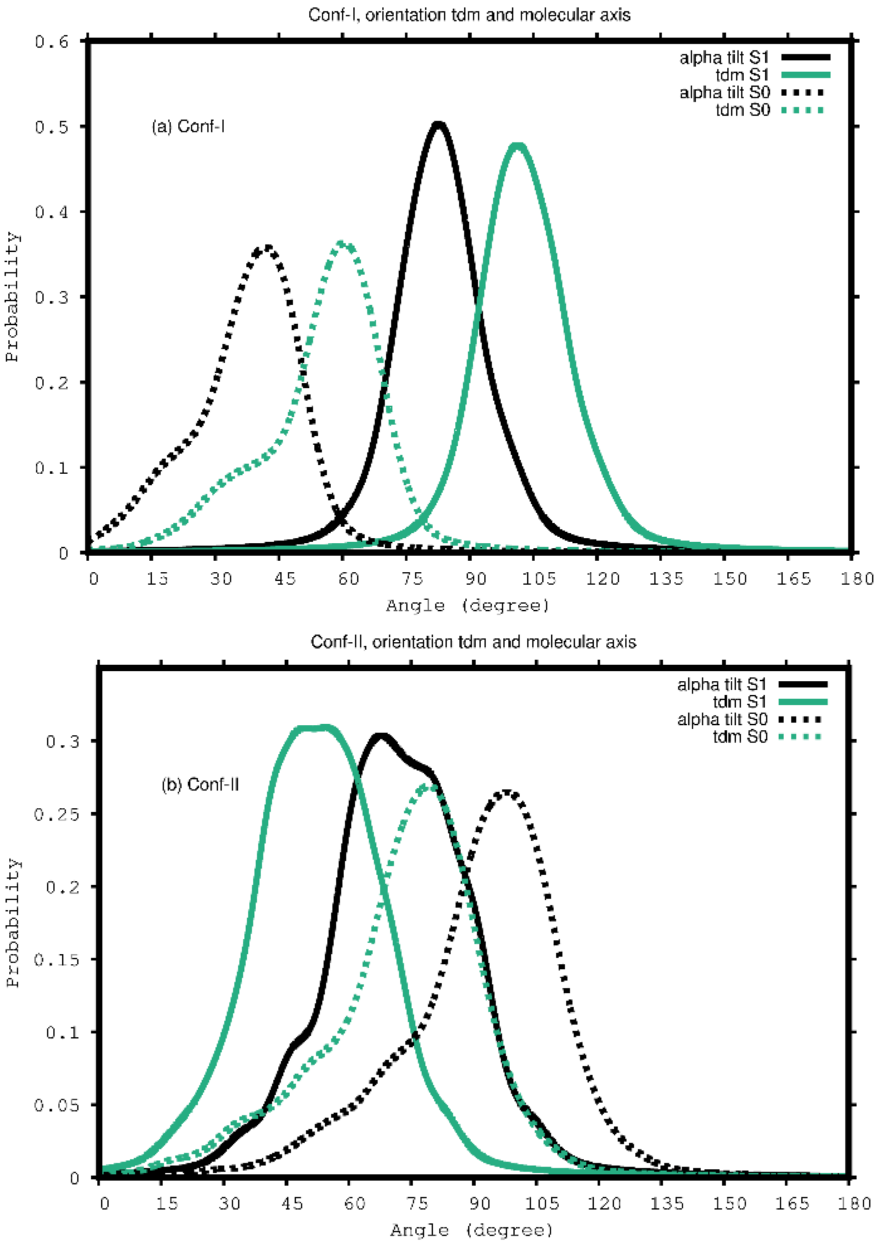

Figure S3 Comparison between the orientations of the tdm with respect to the z-axis and of the α-tilt angle of the first excited and the ground state in DPPC at 298K.

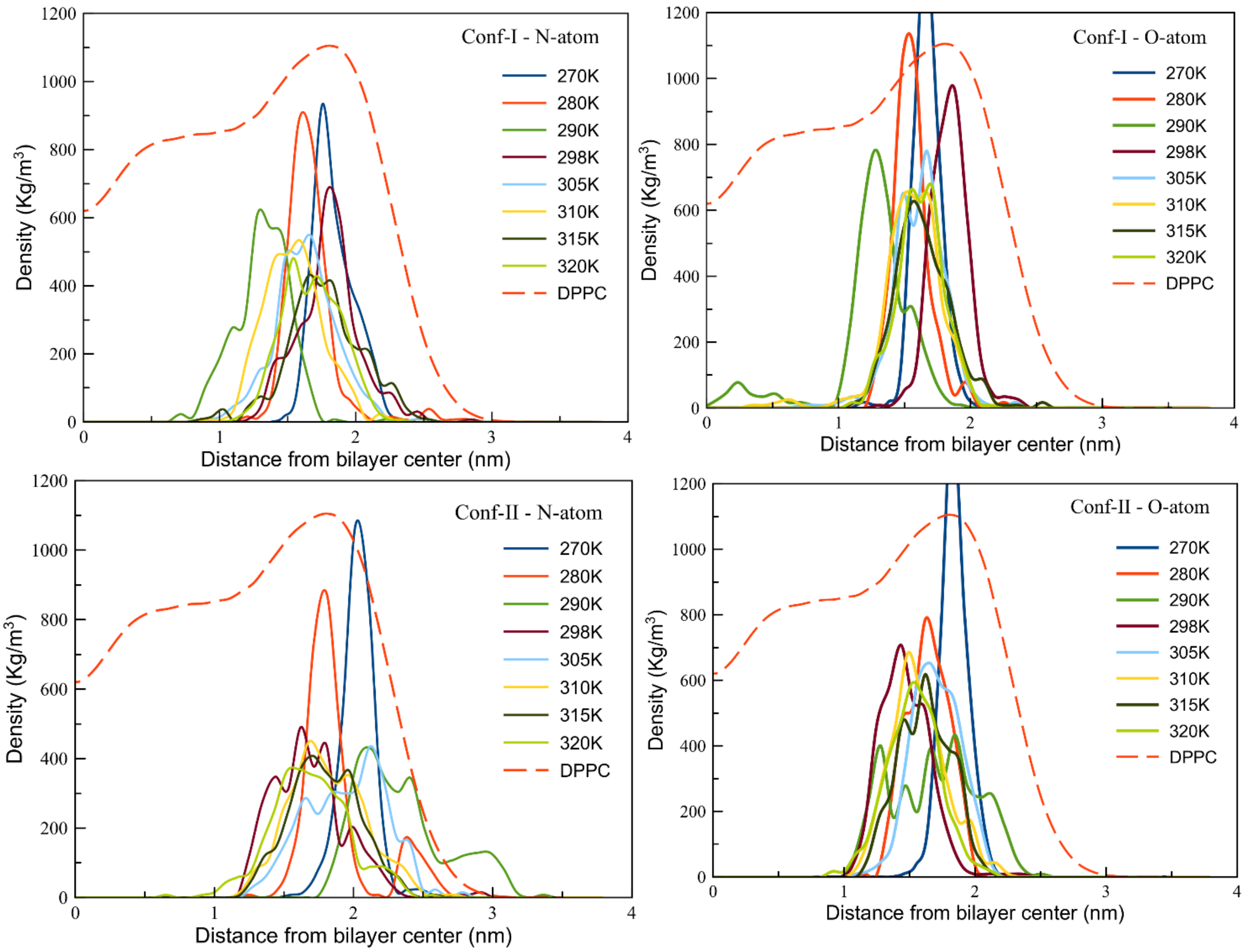

Figure S4 Distribution of the position of the N-atoms (left columns) and O-atoms (right columns) in the headgroup of Laurdan excited in the S_1 state embedded in the DPPC membrane at different temperatures. As a guide to the eye, these densities have been magnified 400 times. The reported DPPC density has been considered for T=305K.

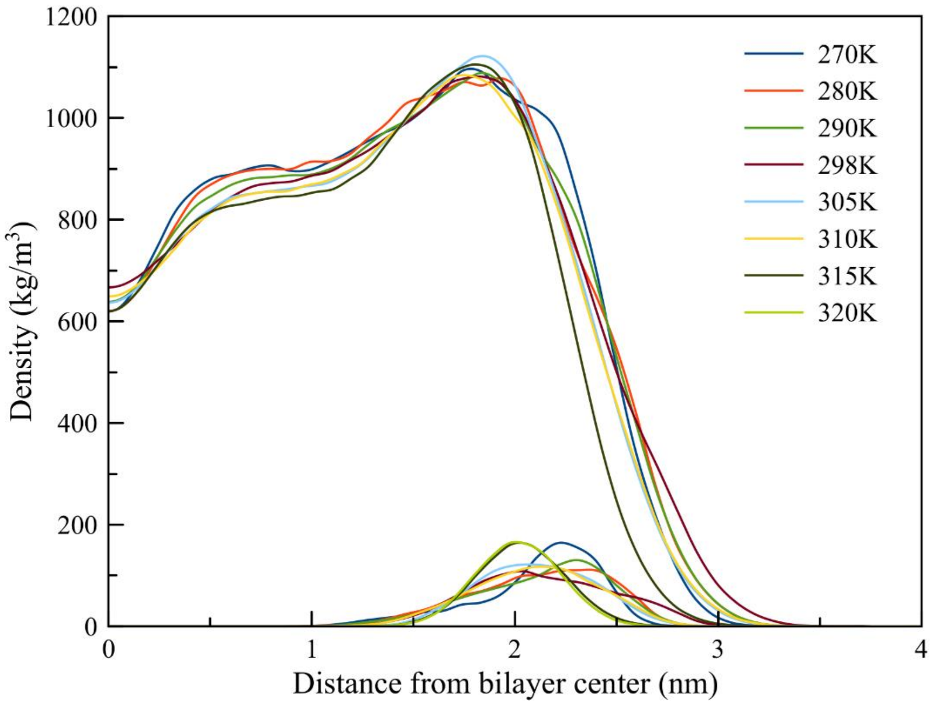

Figure S5 Distribution of the DPPC membrane at different temperatures. The phosphorous atoms distribution is also reported and have been magnified 400 times.

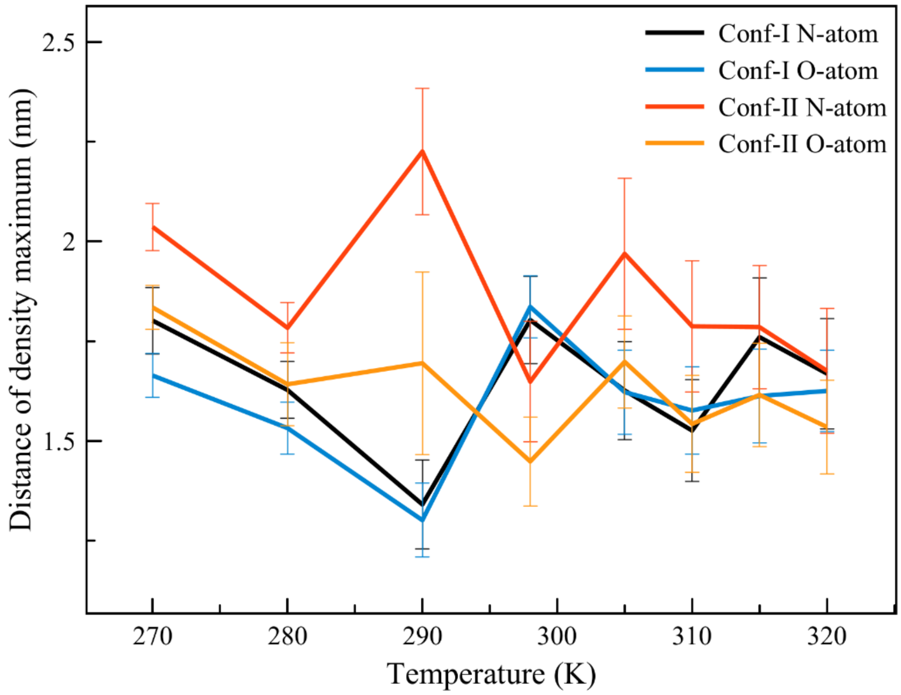

Figure S6 Distance (nm) of the density maxima of the N- and O-atoms in the head group of Laurdan in its S_1 excited state with respect to the center of the DPPC membrane at different temperatures. Error bars have been computed considering HWHM of the distribution plots reported in Fig. S4.

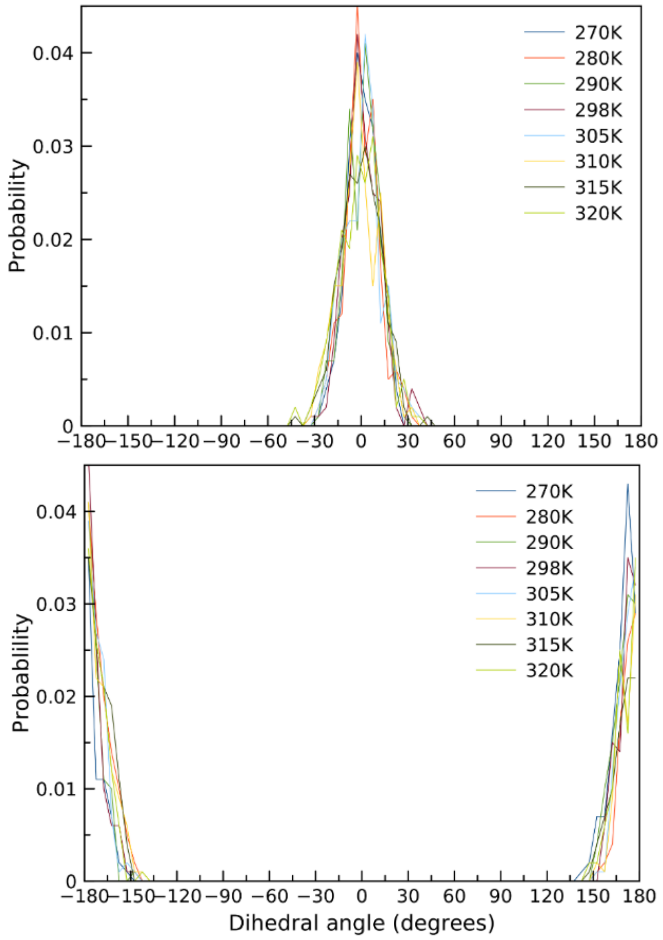

Figure S7 Distribution of the characteristic C-C-C-C dihedral angles of Laurdan embedded in the DPPC membrane at different temperatures, from MD using the charges of the S_1 excited state for Conf-I (top) and Conf-II (bottom).

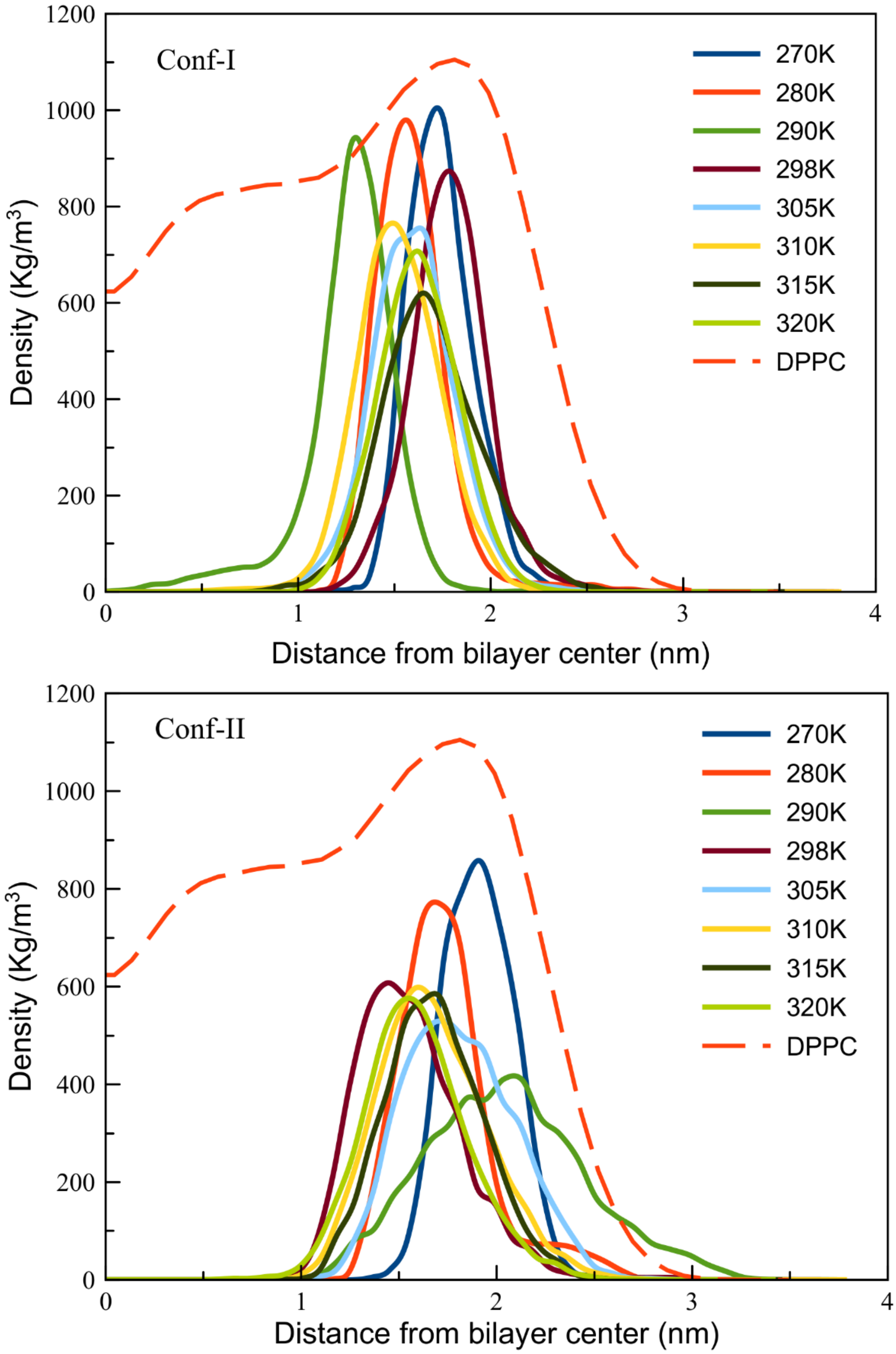

Figure S8 Density distribution of the head group of Laurdan in the DPPC membrane at different temperatures. The calculations have been done using the S_1 excited state for Conf-I (top) and Conf-II (bottom) of Laurdan. The reported DPPC density has been considered for T=305K.

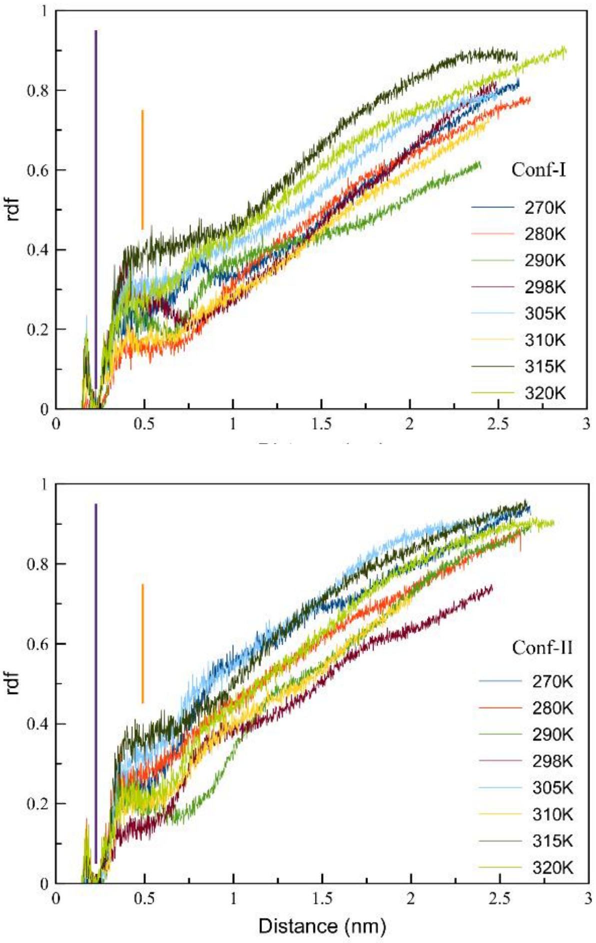

Figure S9 Radial distribution functions of water with respect to the Laurdan head of Conf-I (top) and Conf-II (bottom). The vertical purple and orange lines note the first and second solvation shells, respectively.

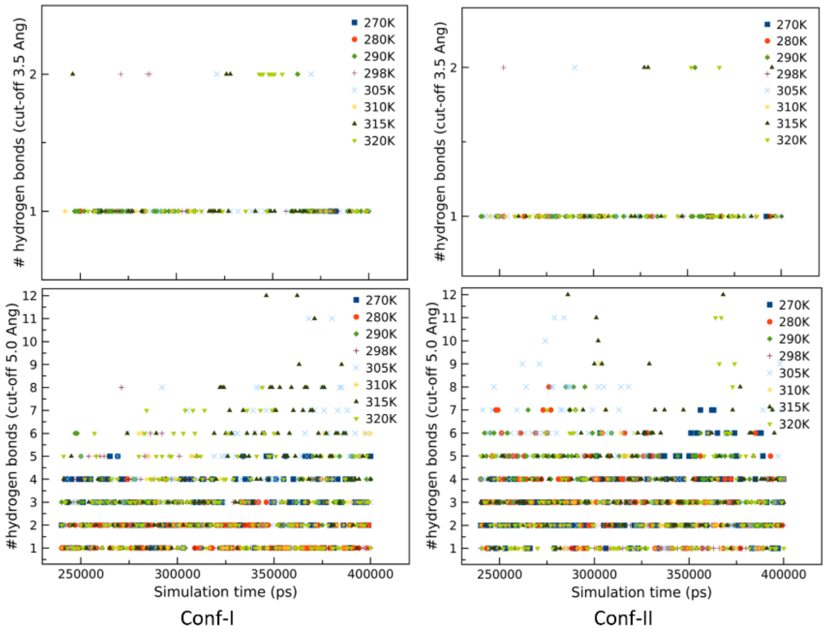

Figure S10 Hydrogen bonds for Conf-I (left) and Conf-II (right) with cut-off at 3.5 Å (top row) and 5.0 Å (bottom row).

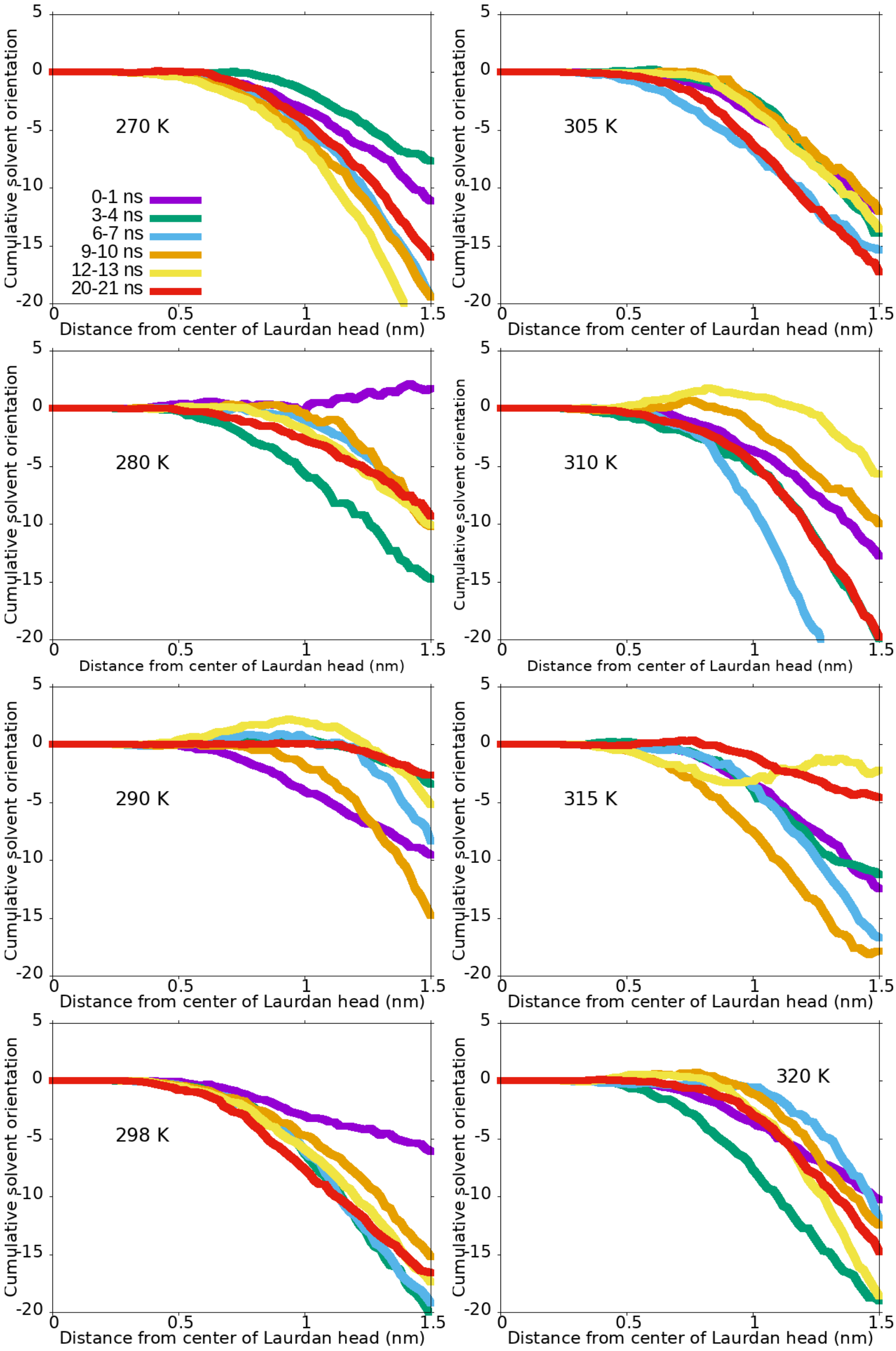

Figure S11 Cumulative solvent orientation as a function of distance from the mass center of the Laurdan head group for Conf-I embedded in the DPPC membrane. The different colors point at the time delay after excitation.

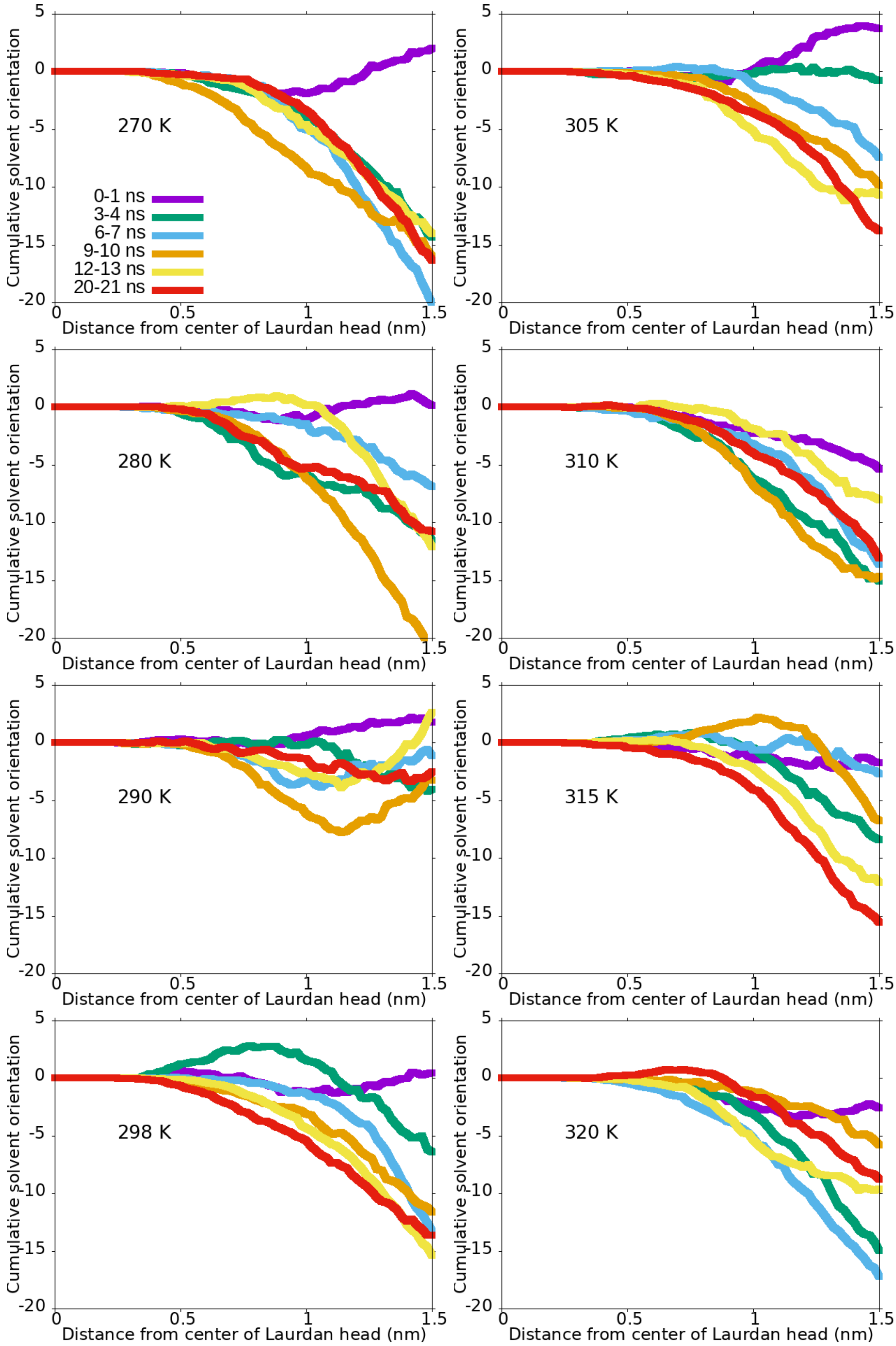

Figure S12 Cumulative solvent orientation as a function of distance from the mass center of the Laurdan head group for Conf-II embedded in the DPPC membrane. The different colors point at the time delay after excitation.

Table S1 Number of hydrogen bonds with cut-off at 3.5 Å and 5.0 Å for Conf-I and Conf-II.

	Cut-off at 3.5 Å		Cut-off at 5.0 Å	
Temp (K)	Conf-I	Conf-II	Conf-I	Conf-II
270	3	3	401	485
280	1	14	241	514
290	58	44	391	611
298	32	10	395	198
305	28	23	583	645
310	15	20	262	301
315	31	29	616	630
320	31	29	450	377

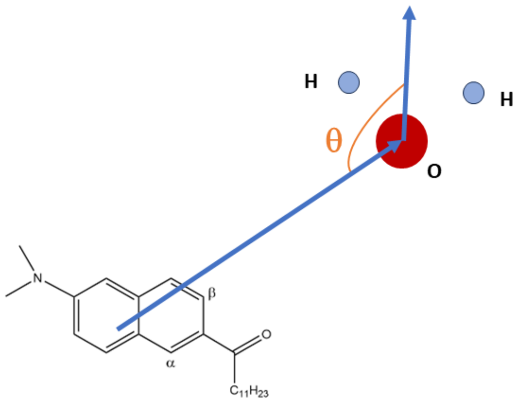

Figure S13 – Depiction of the angle θ between the vector pointing from the center of mass of the Laurdan head group to the oxygen atom of the water molecule, with the vector from this oxygen atom to the middle point between both H atoms of the same water molecule. This angle is used to describe the solvent polarization in the neighborhood of the probe's head.

Table S2 Averaged area per DPPC lipid while increasing the temperature of the membrane for both conf-I and conf-II. Standard deviation is reported in parentheses.[a]

**APL (Å^2)**	**270K**	**280K**	**290K**	**298K**	**305K**	**310K**	**315K**	**320K**
Conf-I	49.84 (0.31)	50.09 (0.44)	49.66 (0.57)	48.76 (0.55)	50.85 (0.50)	51.25 (0.63)	54.55 (1.09)	55.91 (0.79)
Conf-II	50.41 (0.32)	50.44 (0.50)	50.22 (0.32)	52.13 (0.72)	50.27 (0.55)	51.49 (0.75)	55.73 (0.93)	55.99 (0.88)

[a] For the APL analysis, we consider the 240-400 ns interval of the MD simulations

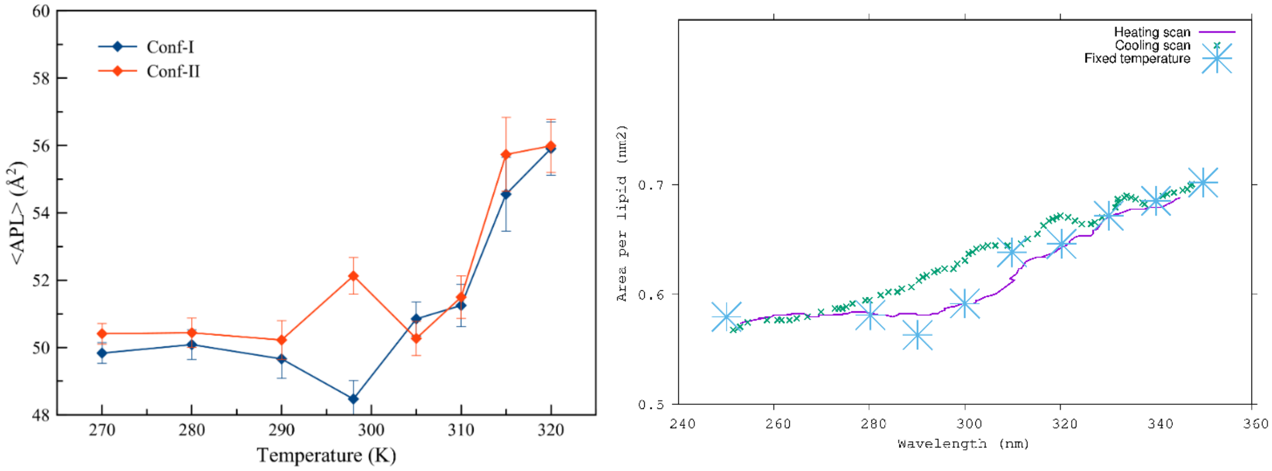

Figure S14 – Comparison between the APLs for the membranes with Conf-I and Conf-II (left) obtained in the current study and the ones for DPPC by Leekumjorn and Sum (right hand side; Biochim. Biophys. Acta A 1768 (2007), 354). Purple solid and green dotted lines correspond to the heating and cooling scan, respectively. Heating and cooling rates of 2.5 K/ns were used to obtain the hysteresis loop. The larger blue stars result from the fixed temperature simulations.

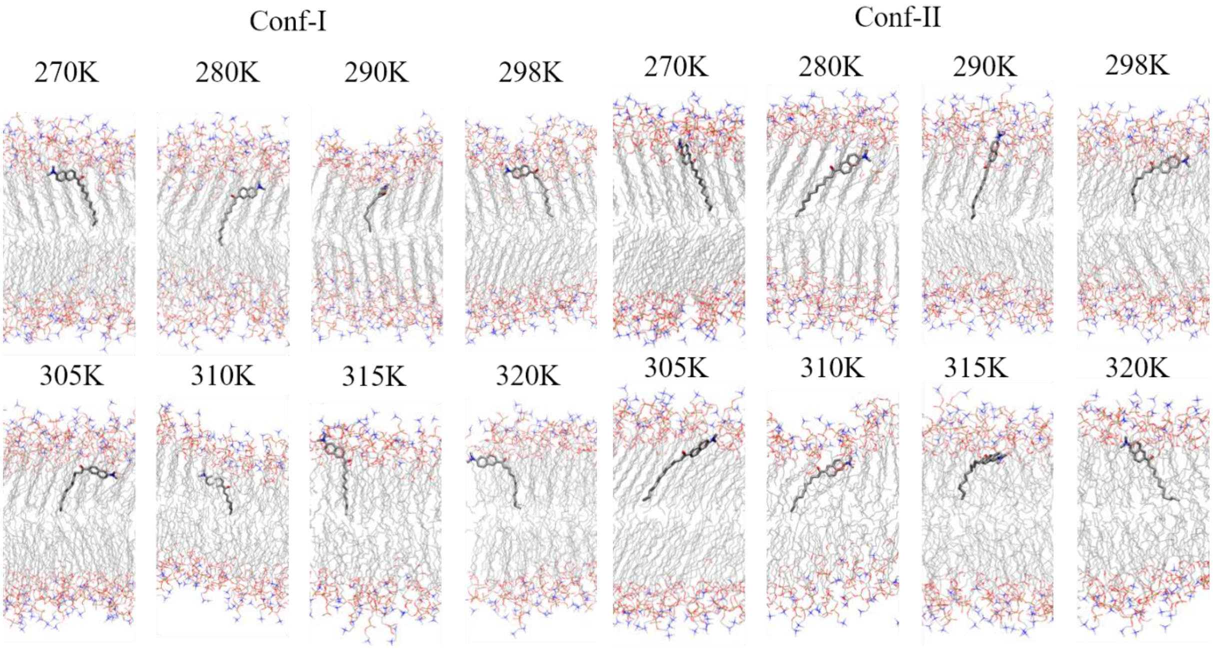

Figure S15 - Representative snapshots of the position of the two conformers of Laurdan embedded in DPPC at different temperatures. The local disruption due to the orientation of the head of laurdan is more prominent for conf-I.

Table S3 Averaged thickness of the membrane (in nm) computed from the position of the P atoms along the trajectory. The standard deviation is indicated in parentheses.[a]

**270**	**280**	**290**	**298**	**305**	**310**	**315**	**320**
4.43 (0.07)	4.62 (0.06)	4.62 (0.05)	4.08 (0.06)	4.18 (0.05)	4.22 (0.05)	3.98 (0.04)	3.95 (0.03)

[a] Comparing our results with the one reported by Leekumjorn, we reproduce the proper trend of decrease thickness while increasing the temperature. Our data also match with the reported values from GROMOS MD simulations of DPPC of 37.2 Å at 323K [J. Phys. Chem. B 2009], 44.1 (1.9) Å at 305K [Biophys. J. 2016, 111, 813-823] as well as experimental value of 42.8 (0.2) Å at 298K.

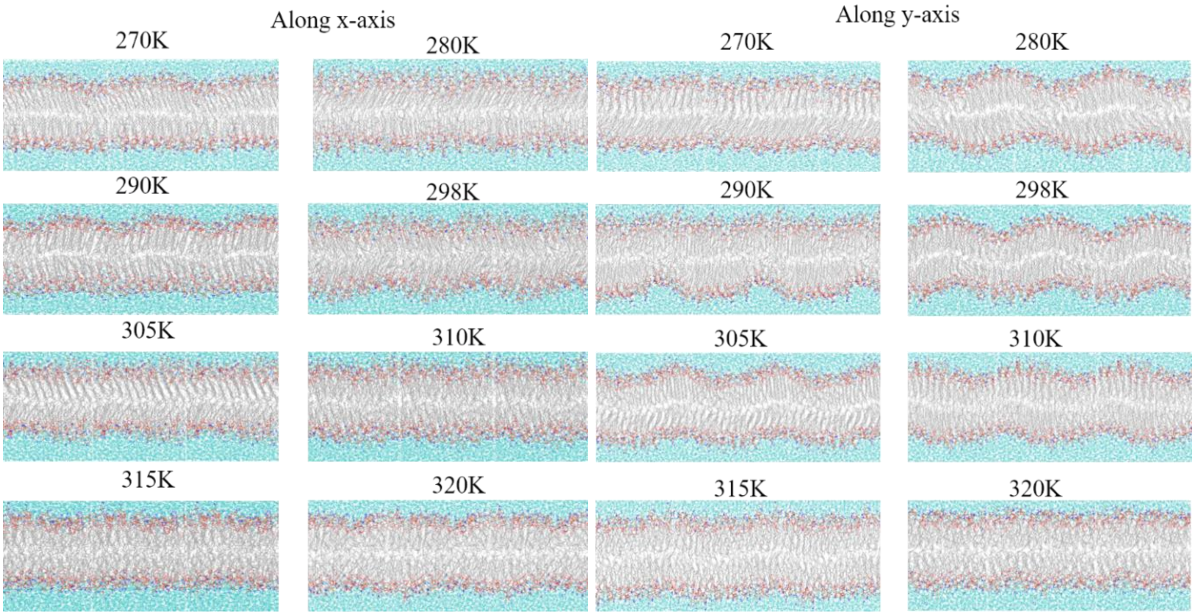

Figure S16 - Representative snapshots of the DPPC membrane taken at different temperatures along both xz- and yz-planes (the z-axis is normal to the bilayer interface).

Analysis of the emission spectra of each conformer

The obtained fluorescence spectra are reported in Figure S17. A clear trend is not visible, but there is a definite change in behavior once the phase transition temperatures are reached and the membrane passes from the crystal (L_c) to the gel ($L_{\beta'}$) and finally the liquid disordered phase (at 314K). To have a clearer vision of the change in maximum emission with increase of temperature, we reported in Figure S18 the behavior of the maximum emission as well as the averaged values (over all the frames). By construction, the values for the former ones are related to one snapshot only. To have a more statistically correct vision, we consider also the latter ones which enables an analysis of the evolution of the averaged emission (from the 50 snapshots) over the different temperatures.

From this plot, it is clear that the two conformers behave in a different way. Especially when the averaged emission values are concerned, Conf-I shows a rather constant emission wavelengths around 350 nm. At 270K, the emission is with a couple of nanometers rather slightly blue shifted. Also at 315K and 320K, the emission can be seen at slightly shorter wavelengths.

The same cannot be said with certainty for Conf-II. In fact, now more fluctuating data are present, probably correlated to the different orientation/position of this conformer in the membrane, which results in being less affected by the environmental changes than Conf-I. In fact, fingerprints of the three membrane phases can be seen. For the crystal phases around 270 and 280K, the averaged Conf-II emission is predicted at 362-368 nm. Contrastingly, at the onset of the gel phase at 290K, a large blue shift is seen. Indeed, Conf-II can rather be found at the outside of the membrane (Figure 4). From the radial distribution functions of Figure S7, it is clear that water is abundantly present in the rather immediate surroundings. Gradually heating towards 305K, the emission peaks shift again further to the red. At the ripple phase $P_{\beta'}$ around 310K, a shift of 15 nm is seen towards the blue. For the L_α phases at 315K and 320 K, emission wavelengths around 360 nm are observed. The course of the peak maxima are further analogous to the one of the averages. For Conf-I, however, a discrimination between the membrane phases based on the energy of the outgoing light is more involved as the energy changes over the various temperatures are rather smooth.

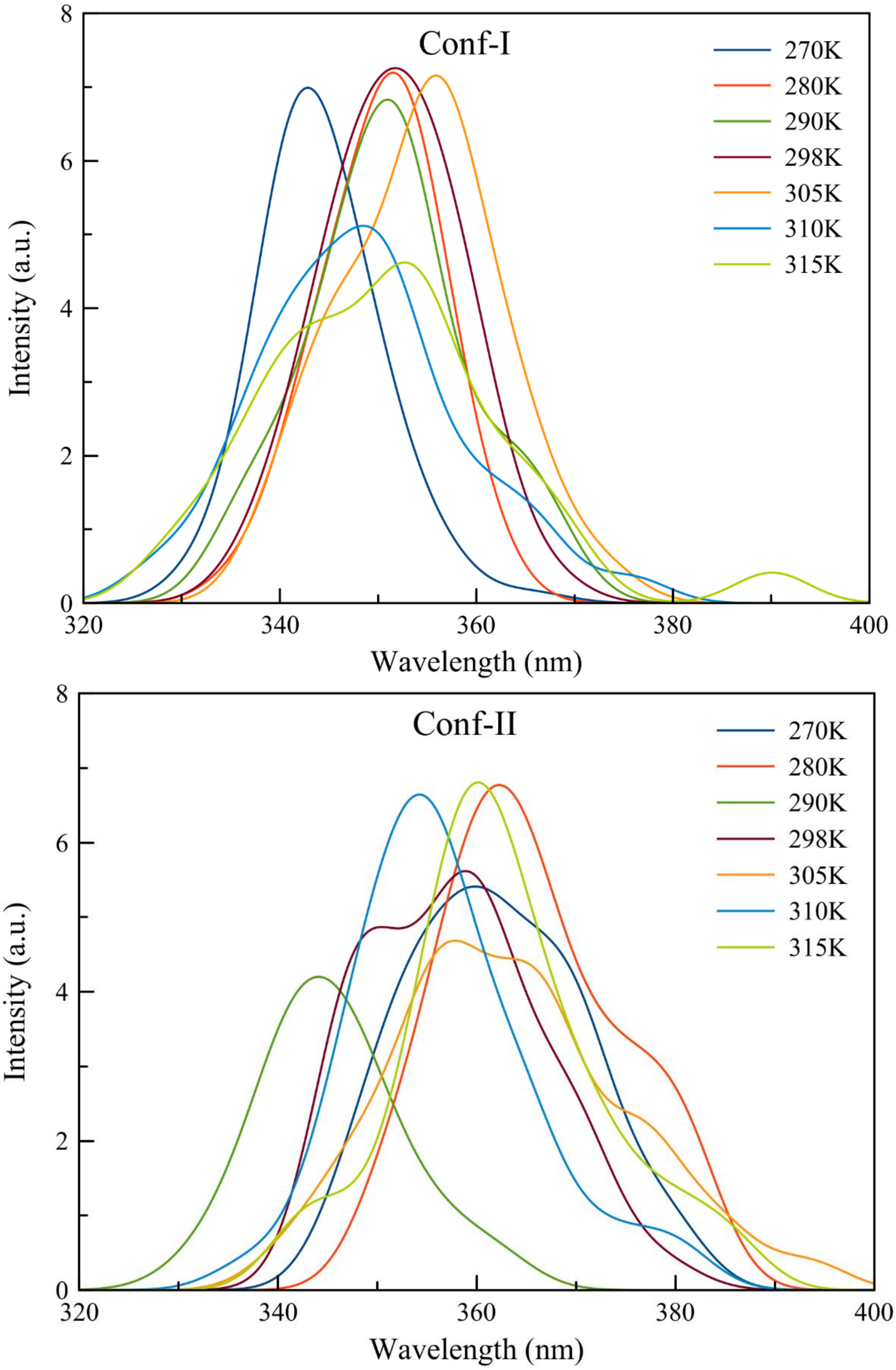

Figure S17 Fluorescence spectra of Laurdan embedded in DPPC at different temperatures.

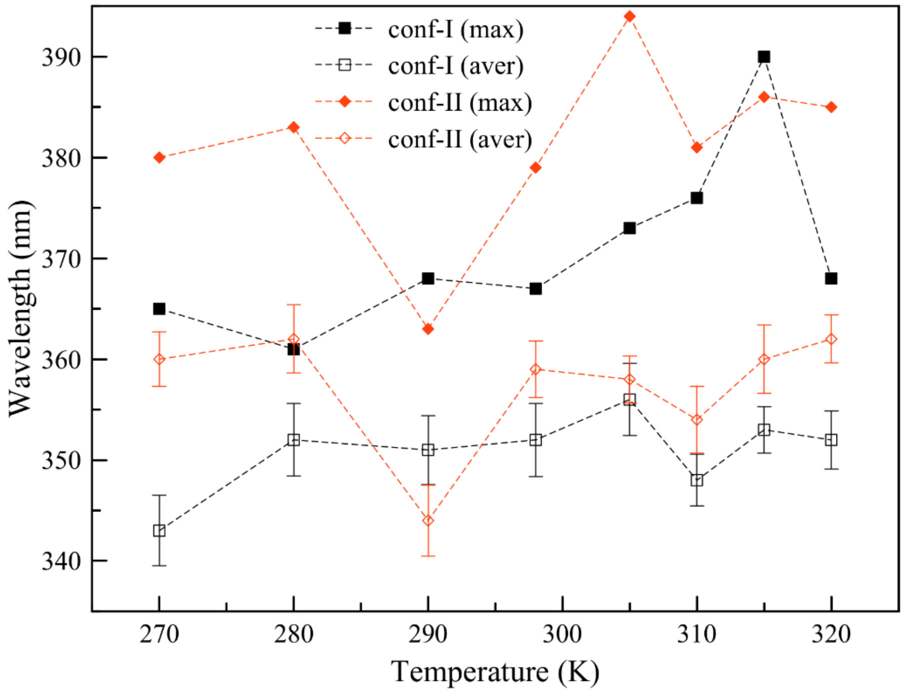

Figure S18 Evolution of the max and averaged emission for the S_1 state with respect to the increase of temperature for both Conf-I and Conf-II. Error bars have been computed considering HWHM of the distribution plots reported in Fig. S13.

Table S4 Detailed analysis of the transitions for the first excited state emission for both conformers at different temperatures.

	**Max wavelength (nm)**	**Aver wavelength (nm)**	**o.s.**	**Lambda**	**transition**
**270-I**	365	343	0.23	0.65	H → L
**270-II**	380	360	0.29	0.64	H → L
**280-I**	361	352	0.25	0.63	H → L
**280-II**	383	362	0.32	0.63	H → L
**290-I**	368	351	0.28	0.63	H → L
**290-II**	363	344	0.17	0.67	H → L
**298-I**	367	352	0.30	0.63	H → L
**298-II**	379	359	0.29	0.64	H → L
**305-I**	373	356	0.31	0.61	H → L
**305-II**	394	358	0.29	0.64	H → L
**310-I**	376	348	0.27	0.63	H → L
**310-II**	381	354	0.29	0.65	H → L
**315-I**	390	353	0.30	0.63	H → L
**315-II**	386	360	0.30	0.63	H → L
**320-I**	368	352	0.28	0.62	H → L
**320-II**	385	362	0.29	0.64	H → L

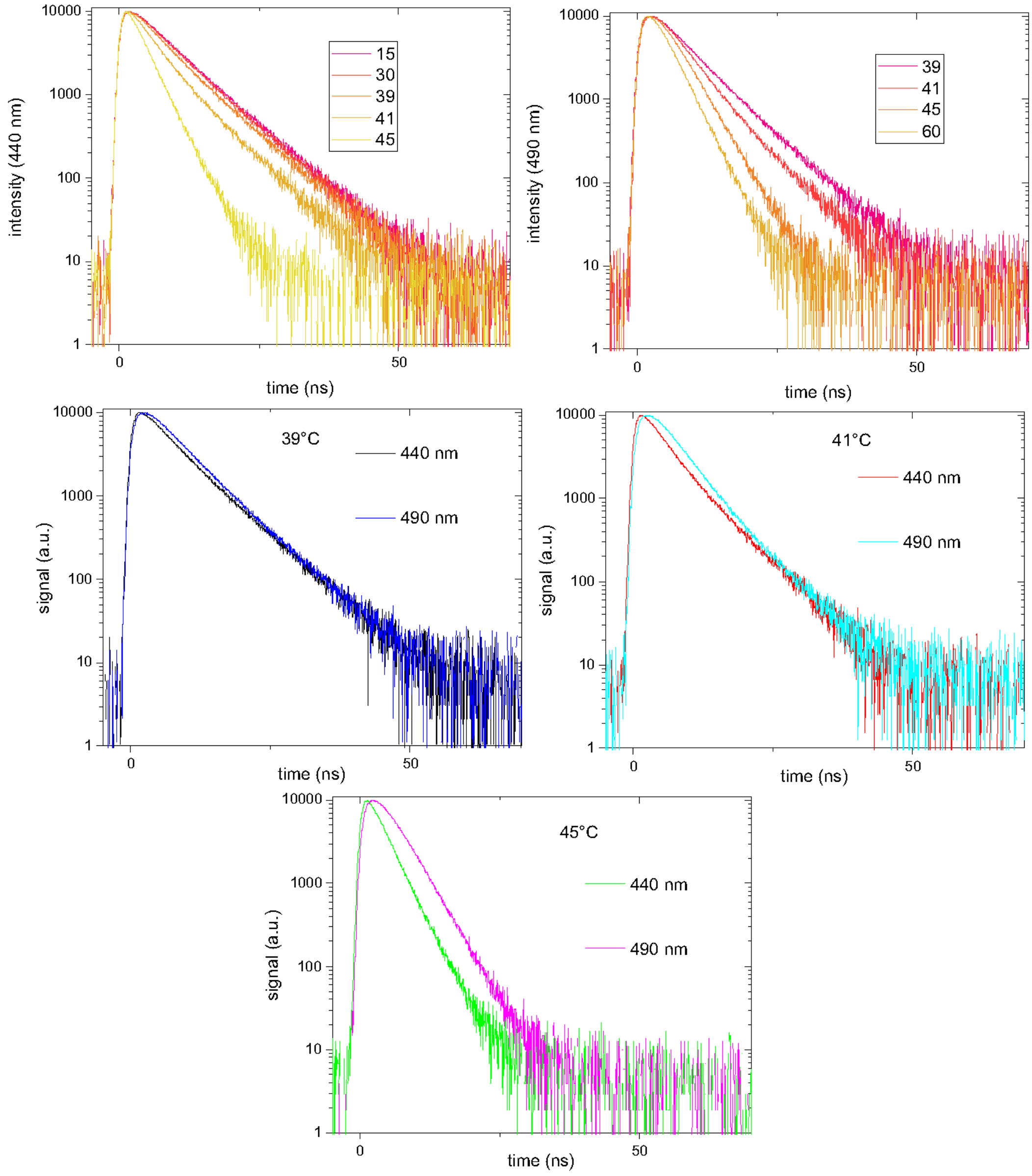

Figure S19 Experimentally obtained emission decay curves for Laurdan obtained at 440 nm and 490 nm at different temperatures (given in °C).

Table S5 Fitted time constants and coefficients for the anistropy decay of Laurdan at 440 nm and 490 nm (see Figure 8).

°C	440 nm (time – ns)	440 nm (static)[b]	440 nm (decay)	490 nm (time – ns)	490 nm (static)	490 nm (decay)
15	3.61 ns	0.26	0.05			
30	2.18 ns	0.25	0.06			
39	1.46 ns	0.23	0.13			
41[a]	1.39 ns	0.18	0.21	2.70	0.09	0.25
45				3.39	0	0.37
60				2.41	-0.01	0.36

[a] For 41°C, the fit has been performed on the first slope of the decay curve only.

[b] Time resolved anisotropy curves were fitted with the sum of an exponential decay and a static contribution. The amplitude of the exponential decay signifies the free moving space with the indicated correlation time. The static fraction on the other hand characterizes orientation which are not reached and is closed linked to the order (parameter) of the system.

Annex two component time resolved anisotropy

Each component contributes to the anisotropy signal by:

$$a_i(t) = A\left((1 - s_i)10^{-\frac{t}{\tau_i^a}} + s_i \right)$$

With the anisotropy of the static molecules A, the fraction of restricted motion s_i, and the correlation time τ_i^a of the motion. The contribution is weighted with the signal intensity at time t:

$$\rho_i(t) = I_i 10^{-\frac{t}{\tau_i}}$$

With the fluorescence lifetimeτ_i,and the intensity I_i. The results are weighted as

$$a_{tot}(t) = \frac{\sum_i a_i(t)\rho_i(t)}{\sum_i \rho_i(t)}$$

The intensity I_i is related to the integrated intensity by $I_i^{int} = 2.3\, I_i \tau_i$.

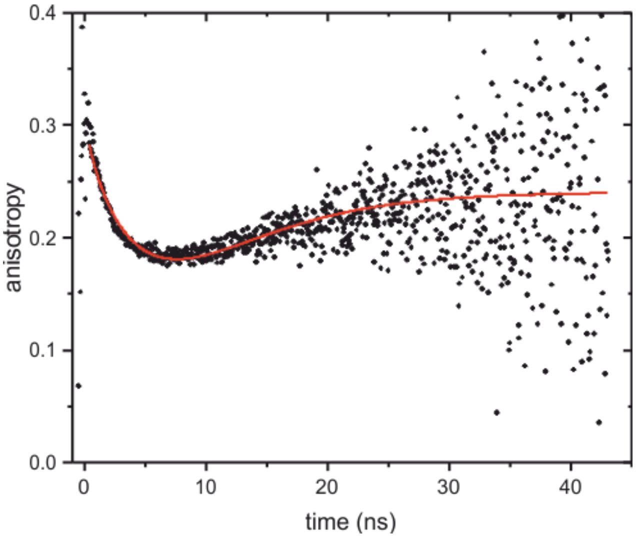

Figure S20: simulation of the time resolved anisotropy recorded at 440nm and 41°C. $\tau_1 = 3.58\, ns,\ \ \tau_2 = 7.46\, ns,\ \ s_1 = 0.23, s_2 = 0.79,\ \ \tau_1^a = 1.7ns, \tau_1^a = 4.6ns,$ A=0.3 and 57% integrated signal of component 1 (corresponding to 73% of time resolved signal of component 1).

Table S6 – S1 Charges of Conf-I and Conf-II used for the MD simulations

	Conf-I	Conf-II
C1	0.149987	0.090783
C2	-0.063341	-0.054328
C3	-0.141456	-0.104891
C4	0.198368	0.120561
C5	-0.144354	-0.099696
N1	0.100849	0.143498
C6	-0.192474	-0.090797
C7	-0.178828	-0.233771
C8	0.105220	0.059247
C9	-0.195617	-0.153508
C10	0.254657	0.204509
C11	0.544605	0.624376
O1	-0.589656	-0.582874
C12	-0.033513	-0.119036
C13	0.097138	0.075498
C14	0.077406	0.075622
C15	0.000553	0.032927
C16	0.020832	0.044544
C17	-0.019263	-0.053545
C18	0.024202	0.028150
C19	-0.044699	-0.025183
C20	0.085423	0.071581
C21	-0.092458	-0.097457
C22	0.022053	0.034643
C23	0.106697	0.098978
C24	-0.092333	-0.089833
